# Comparing the cost effectiveness of nature-based and coastal adaptation: A case study from the Gulf Coast of the United States

**DOI:** 10.1371/journal.pone.0192132

**Published:** 2018-04-11

**Authors:** Borja G. Reguero, Michael W. Beck, David N. Bresch, Juliano Calil, Imen Meliane

**Affiliations:** 1 Institute of Marine Sciences, University of California, Santa Cruz, California, United States of America; 2 The Nature Conservancy, Santa Cruz, California, United States of America; 3 Swiss Federal Institute of Technology, ETH Zurich, Zurich, Switzerland; 4 Department of Ocean Sciences, University of California, Santa Cruz, California, United States of America; Universidade de Vigo, SPAIN

## Abstract

Coastal risks are increasing from both development and climate change. Interest is growing in the protective role that coastal nature-based measures (or green infrastructure), such as reefs and wetlands, can play in adapting to these risks. However, a lack of quantitative information on their relative costs and benefits is one principal factor limiting their use more broadly. Here, we apply a quantitative risk assessment framework to assess coastal flood risk (from climate change and economic exposure growth) across the United States Gulf of Mexico coast to compare the cost effectiveness of different adaptation measures. These include nature-based (e.g. oyster reef restoration), structural or grey (e.g., seawalls) and policy measures (e.g. home elevation). We first find that coastal development will be a critical driver of risk, particularly for major disasters, but climate change will cause more recurrent losses through changes in storms and relative sea level rise. By 2030, flooding will cost $134–176.6 billion (for different economic growth scenarios), but as the effects of climate change, land subsidence and concentration of assets in the coastal zone increase, annualized risk will more than double by 2050 with respect to 2030. However, from the portfolio we studied, the set of cost-effective adaptation measures (with benefit to cost ratios above 1) could prevent up to $57–101 billion in losses, which represents 42.8–57.2% of the total risk. Nature-based adaptation options could avert more than $50 billion of these costs, and do so cost effectively with average benefit to cost ratios above 3.5. Wetland and oyster reef restoration are found to be particularly cost-effective. This study demonstrates that the cost effectiveness of nature-based, grey and policy measures can be compared quantitatively with one another, and that the cost effectiveness of adaptation becomes more attractive as climate change and coastal development intensifies in the future. It also shows that investments in nature-based adaptation could meet multiple objectives for environmental restoration, adaptation and flood risk reduction.

## Introduction

Natural hazards in coastal zones pose high and increasing risks to people, property and habitats [1]. The combined influence of coastal storms, rising sea levels, urban development, population growth and land subsidence are increasing flood risk in coastal areas worldwide [2–7]. By 2050, flood damage in the world’s coastal cities is expected to reach $1 trillion a year [8]. As sea level rises, tropical cyclones will pose a greater risk of extreme flooding and are likely to inflict the greatest damages on highly populated shorelines [4]. In the United States (US), climate change and the rise in sea-levels will impact many economic sectors [9], threaten people [10] and loss of historic and prehistoric archaeological sites and many other cultural assets [11]. Globally, it is projected that coastal growth in population and development will outpace progress in risk reduction [12].The need to upgrade existing flood protection and to plan for future coastal risks is becoming increasingly apparent, but the costs may be daunting [13–15]. However, effective adaptation requires understanding the different drivers of risk from an economic perspective, including coastal development and the impacts of climate change [16,17].

Countries are undertaking big investments to address current and future flood risks. Many USA states and federal agencies (e.g. US Army Corps of Engineers or US Departments of Transportation) invest significant in hazard mitigation. For example, the Federal Emergency Management Agency (FEMA) spends $500 million annually in pre-hazard mitigation to reduce flooding hazards. Yet the majority of these hazard mitigation and adaptation funds are destined for the creation of “grey infrastructure”, i.e. built structures such as seawalls, which can further degrade coastal ecosystems [18,19]. Today, approximately 14% of the US coastline has been armored [20]. Meanwhile, coastal habitats that provide an important first line of defense continue to be lost, thus further exposing people and property to coastal hazards [21,22].

Nature-based, green or natural infrastructure is emerging as a cost-effective option to reduce the impacts of storm surge and waves [23]. Nature-based measures use natural features of ecosystems for coastal protection (e.g., wetland restoration). A growing body of knowledge and experience supports their effectiveness for coastal defense [23–26]. Coastal ecosystems like coral reefs, oyster reefs, mangroves and salt marshes protect the coast by reducing wave energy, trapping sediments, and attenuating storm surge [27–33]. Nature-based measures also offer a dynamic solution to challenges such as sea level rise, because ecosystems may adapt to, and grow with, their changing environment [34–36].

There is also growing interest among policy-makers. For example, the European Union’s Biodiversity Strategy urges the implementation of green infrastructure as an investment priority for sustainable growth for 2020, and encourages that natural processes become a systematic part of spatial planning [37]. Similarly, the EU Research and Innovation policy agenda on Nature-Based Solutions and Re-Naturing Cities aims to lead to more sustainable and resilient societies, recommending the development and deployment of Nature-Based Solutions that maximize cost-effectiveness and co-benefits [38]. In the United States, there are also increasing calls for the development of resilient infrastructure, such as the use of natural ecosystems to both sequester carbon and adapt to the effects of climate change [39]. The alignment of restoration and climate adaptation is receiving greater federal attention and recognition by coastal planners [40].

However, the optimum solutions for adaptation are unlikely to be exclusively green *or* grey, but rather a diverse portfolio of options including green (e.g., wetland or dune restoration), grey (e.g. seawalls, and breakwaters) and policy (e.g., land use zoning) measures [41,42]. This requires direct comparison with one another and assessment of their costs and benefits [38]. There is also a need for better strategic visions for risk reduction and climate adaptation that involves specific science to address stakeholder concerns and supports climate change policy [16,43]. Analyses should be able to: (i) identify areas most at risk, (ii) quantify losses and damages under various present and future scenarios, and (iii) compare and prioritize potential solutions with cost-benefit analysis [17,44,45]. The information and tools necessary to analyze and prioritize adaptation measures have been scarce and limited to local sites such as estuaries, islands and bays and included only a limited number of alternatives [46–49], although they are needed at many planning scales (e.g., Louisiana Coastal Master Plan, [50–52]). Furthermore, while nature-based solutions are gaining momentum as an adaptation strategy, the lack of explicit quantification of its cost and effectiveness is inhibiting widespread application [33,40]. This study helps address these gaps.

This paper crucially addresses the need for direct comparisons of the cost effectiveness of green, grey and policy adaptation measures for current and future risks. We assess risks and adaptation measures across the US Gulf Coast; a region that is home to three of the top five US metro areas by exposed assets and has experienced substantial coastal development in the recent decades [53–55]. The region faces intense hurricanes, and considerable land subsidence and sea level rise [56–60]. In this study, we consider a diverse set of scenarios, drivers and timeframes to provide a robust and flexible framework with which to measure risk, its drivers, and the risk reduction potential of a diverse adaptation portfolio, including both green and grey measures. This paper will help answer the crucial questions: (i) what is at risk (and what is the cost of doing nothing), and (ii) what are the cost and benefits of different adaptation strategies.

## Methods and data

**Fig 1:**
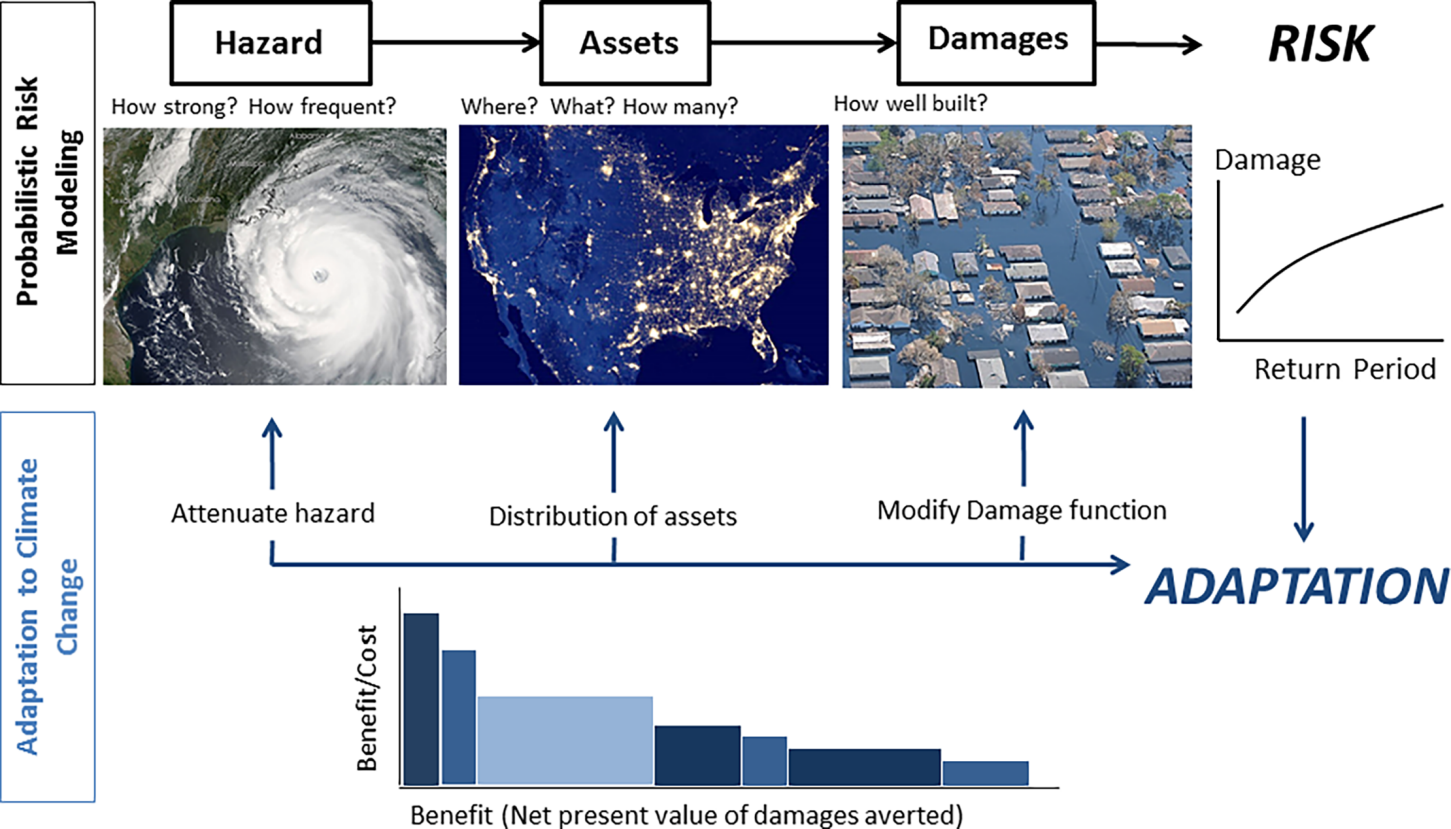
Methodology. Workflow and methodological approach for the quantitative assessment of (i) probabilistic risk (first level, in black) and (ii) the economics of adaptation measures (second level, in blue). Risk is formed from three terms: the hazard (flooding from a storm), the distribution of assets (value and property exposed to the flood), and the damages inflicted on them (calculated using damage functions for each asset type). The risk is defined quantitatively by losses and damages associated to probability (or return periods). Adaptation measures can be calculated from their effect on each risk term and compared in a cost-benefit analysis. A benefit-cost curve (graphic in lower panel) ranks different adaptation measures by decreasing benefit to cost ratios (vertical axis, height of the bars) and represents the benefit as the net present value of the damages averted when the measure is implemented (horizontal axis, width of the bars). Sources of images: (left and middle) Earth Observatory, National Aeronautics and Space Administration, and (right) National Oceanic and Atmospheric Administration/Department of Commerce, photo credit: reprinted from Lieut. Commander Mark Moran, NOAA Corps, NMAO/AOC under a CC BY license, with permission from NOAA, original copyright 2005.

To assess the cost effectiveness of various adaptation measures, current and future risks of coastal flooding along the US Gulf Coast is assessed following the Economics of Climate Adaptation (ECA) framework [61–63]. Fig 1 represents this approach where risk occurs at the intersection of economic assets and the hazard of coastal flooding (upper part in the Figure), and adaptation can have an effect of each component of risk (lower part). The analysis took three steps: (i) assess baseline or current risk (i.e. the probability of losses today); (ii) estimate future risk; and (iii) compare cost and benefits of adaptation measures. As applied here, the ECA framework is implemented comprehensively in the free open-source software ‘*CLIMADA’* [64], and its ‘*COASTAL’* module [65]. To enhance the reproducibility of the results and methods (e.g., [66]), a research protocol with steps and datasets have been made available online [67], and the source code can be accessed through github [64,65]. The main steps of the analysis are explained in detail below.

### Assessing current risk

Risk is quantified as a loss associated with a certain probability (e.g., [68]). The event loss or damage is the sum of all individual losses resulting from a single occurrence of a natural hazard (e.g. floods). Each individual loss is quantified from three terms (Fig 1):

- **Hazard** (or ‘peril’): defined by the location, frequency and intensity of events (storm flooding), i.e. where, how often and with what intensity do storms occur?
- **Assets exposed**: defined by the location and value of the distinct types of buildings and assets.
- **Damages to assets**: is the relationship between the extent of damage and the event intensity, defined by damage (or vulnerability) curves.

**Fig 2:**
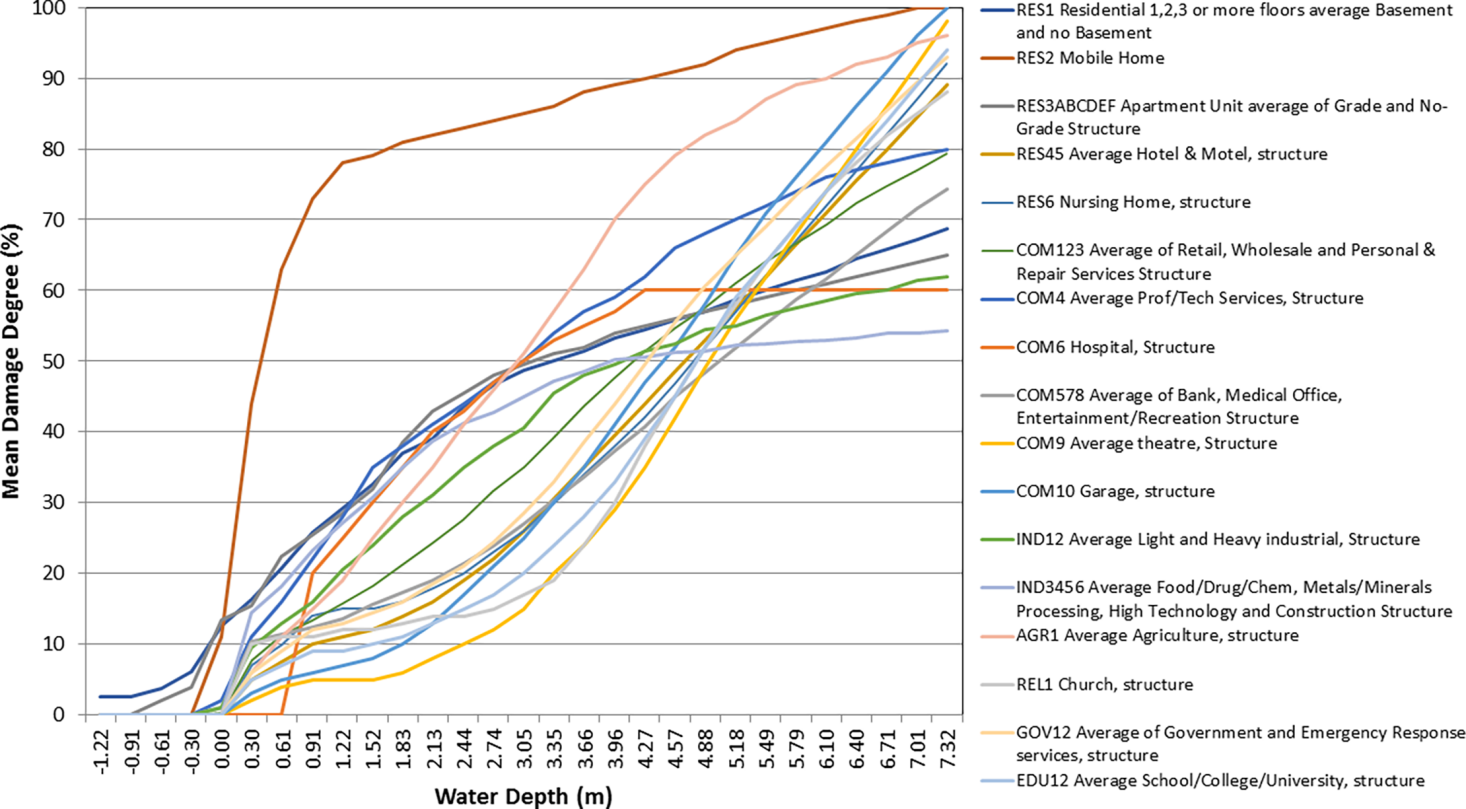
Damage functions. Damage functions to infer damages for the different asset types (in legend). The curve relates water depth (horizontal axis) to the mean damage degree to the asset value or percentage damaged (vertical axis).

**Fig 3:**
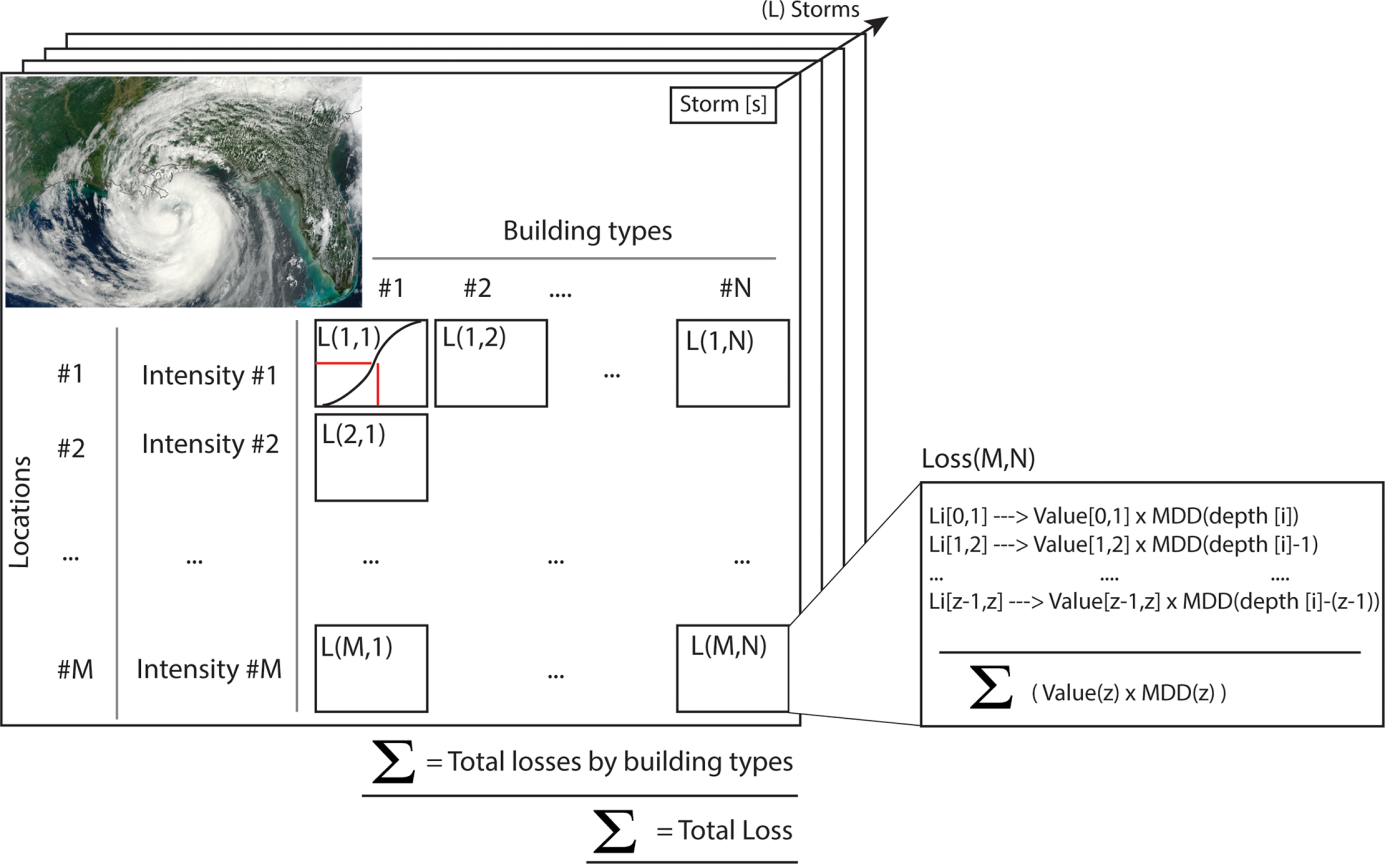
Risk implementation. Pseudo code implementation of the probabilistic risk assessment of coastal flooding. For each storm, the loss is calculated considering assets at each elevation, across locations and types of building or assets. The loss at each elevation, Li(z-1,z), is calculated multiplying the value of the property by the Mean Damage Degree (MDD), as calculated from the damage curves and the flooding depth (see Fig 2). The box in the right panel shows a pseudo code for the loss (L) at one location (M) for a specific asset or building type (N). For example, for a storm flooding at location M with a 40% MDD and an asset value of $1 million, the resulting loss would be 400,000 $. Sources of photo: Earth Observatory, National Aeronautics and Space Administration.

In situations with limited observations, like flooding risk from hurricanes, stochastic simulations are recommended for assessing risks [69,70]. A “probabilistic” or “stochastic” risk assessment simulates a set of possible events that could occur during a period of time (i.e. thousands of events) and which are informed by the historical distribution of storms. In this study, we simulate a probabilistic set of storms and calculated flooding and damages from them. The process is summarized into the following steps:

1. The historical distribution of storms (from 1851 to the present, taken from http://weather.unisys.com/hurricane) is used to generate a probabilistic set of *15*,*000 storms* using random walks with random origin and track pathways. For details on the storm simulations, see [71] and S1 Fig that shows a comparison with the historical distribution for the Accumulated Cyclone Energy and the number of storms.
2. For every storm, the pressure, wind, rainfall, wind-waves and storm surges fields are calculated using parametric models (see Supporting Information for details on the models). The flooding extent is computed from the total water level, as the combination of mean sea level, tides, surges and wave-induced run up [72].
3. For each study unit, we calculate the exposed assets by ground heights (e.g. value of property between topographic elevation 0 and 1 meters, 1 and 2, etc.) because, to calculate damages at each site, flooding needs to be expressed relative to the ground elevation and the bottom of the building. For this we use a national Elevation Dataset [73] and asset values from the HAZUS database at a block level [74], which are aggregated by census tracts and integrated into a total of 3,238 units (S4 Fig).
4. For each asset type (i.e. type of building), a Mean Damage Degree (MDD) is calculated for each relative water depth at each asset location (the difference between flood height and ground elevation) obtained from damage functions. A damage function (Fig 2) provides the damage inflicted by the hazard intensity to the total value of the asset. They were obtained from HAZUS [74] and the original set of 100 curves reclassified into 17 sub-types (Fig 2).
5. The damage or loss is calculated by multiplying the MDD (at each elevation), by the value of the asset. The total loss is the sum of relative losses of all asset types across elevations (see pseudo-code and a graphical description in Fig 3). The loss at location *M* and asset *N* is computed as: *Loss* (*M,N*) = ∑_*z*_ *value*(*z*) * *MDD*(*z*). The sum across sites gives the total damage from one event (i.e. the event damage). For example, in the pseudo code in Fig 3, losses are aggregated by building types and then added to calculate a total damage from the storm: *Total loss from storm S* = ∑_*M* = *Locations*_ ∑_*N* = *Buildings*_ *L*(*M,N*).

This process is repeated for all the types of assets, at all locations, and throughout storms. The result is a statistical distribution of losses, i.e. dollar-value against probability, which characterizes the risk. In this study, we use two statistics to describe risk: (1) the Annual Expected Damage: the average annualized flood losses to property, calculated as the total sum of each event damage multiplied by the probability of each storm; and (2) the 1-in-100-year loss: the flood loss with a 0.01 probability of happening in any given year. All risk values are in US dollars adjusted to the value in year 2015.

### Assessing future risk

Future risk is derived from both changes in climate and in economic exposure. We calculate the future risk for two timelines: years 2030 and 2050 (with reference for risk calculations in 2010). The effects of climate change on the hazards consider: land subsidence, sea level rise and changes in intensity and frequency of storms. Sea level rise is calculated from the historical trends [75], which is regarded as a conservative approach [76]. We also account for land subsidence, a dominant factor in the highly subsiding Mississippi delta, using [77] to calculate relative sea level rise (S3 Fig). We introduce the effects of climate change in storms through changes in intensity and frequency. We consider that: the intensity of all categories of storms increased by 11% by the end of the century; category 4 and 5 hurricanes become 80% more frequent, but the lower intensity storms 28% less frequent; based on available reviews and projections [78–80].

Change in economic exposure is particularly acute in coastal areas due to the intense development in coastal zones. For example, the Gulf Coast population has increased by 109% since 1970, compared to a 52% increase in the US total [55]. We considered two economic exposure growth scenarios to describe increases in the total future exposure: (1) ‘Low’: assumes an annual compound growth in the value of existing assets of 1% per year; and (2) ‘High’: assumes a 2% annual growth. These rates to estimate future asset value were set based on [54,55,81]:

1. past historical data on the House Price Index, by state, which ranges from 21.1% in Mississippi to 43.7% in Louisiana, with an average of 2.67% for the region from 2001–2010;
2. historical evolution of economic growth, and
3. projections from the World Bank and PwC Economics [81] for the years 2030 and 2050, which correspond to annual rates of 2.89% and 3.89%, respectively.

For the sake of simplicity, the results section focuses on the results and analysis of the ‘Low’ economic exposure growth scenario. The comparison with the other scenario is discussed when relevant, but detailed results for the high growth scenario can be found in the Supporting Information.

The risk in the future is calculated by combining the future exposure (asset value) with the future climate. However, to better understand how risk will increase, we assess the contribution of economic development and climate change independently, by calculating the risk in the future with: (i) the future exposure and present hazards and (ii) present exposure and future hazards. For example, we calculate present risk with present exposure (E2010) and present hazards (H2010), and future risk in the year 2030 with the exposure in 2030 (E2030) and the storms and sea level rise by 2030 (H2030). The contribution of economic development is calculated using (E2030, H2010), and the contribution of climate with (E2010, H2030).

### Assessing the economics of adaptation

Assessing the cost (i.e. construction and maintenance) and benefits (i.e. losses averted) of adaptation is challenging. The potential damage averted from each adaptation measure is particularly uncertain, even for those for which extensive research and experience exists, such as improved building codes or seawalls. Furthermore, the performance of each measure depends on local characteristics. However, across the Gulf, we aim to compare large-scale adaptation strategies as a first quantitative cut. The method (as coded in the open-source model ‘*CLIMADA*’ [71]) can be summarize into the following steps:

1. Estimate the benefit of each measure: each measure at specific locations is assumed to protect property for a certain period of time (for which the benefit is calculated).
2. Calculate the cost of each measure: includes the cost of construction (depends on dimensions of the measure and unitary costs) and the cost of regular maintenance.
3. Calculate Net Present Value (NPV) of costs and benefits: the difference between the present value of cash inflows (benefits from adaptation) and the present value of cash outflows (cost and maintenance throughout the implementation period). NPV is calculated as follows:
  - Calculate baseline risk (today) with and without the measure: calculate annual expected damage with no measures and with the effect of the measures applied; the difference is the benefit of implementing the measure today.
  - Calculate future risk (e.g. in year 2030): using future assets and expose future hazards, calculate annual expected damage with no measures and with the measures applied; the difference represents the future benefit of the measure.
  - Discount the benefit to present terms: discounting benefits for a total of T years, its NPV will be: $NPV=\sum_{t=1}^{T}\frac{b_{i}}{{\left(1+i\right)}^{t}}$, where *i* is the discounting rate, used in economic analysis to consider productivity of capital and the preferences of the population.
  - Discount the cost to present terms: as for benefits.
4. Calculate the benefit to cost ratio for each measure.

**Fig 4:**
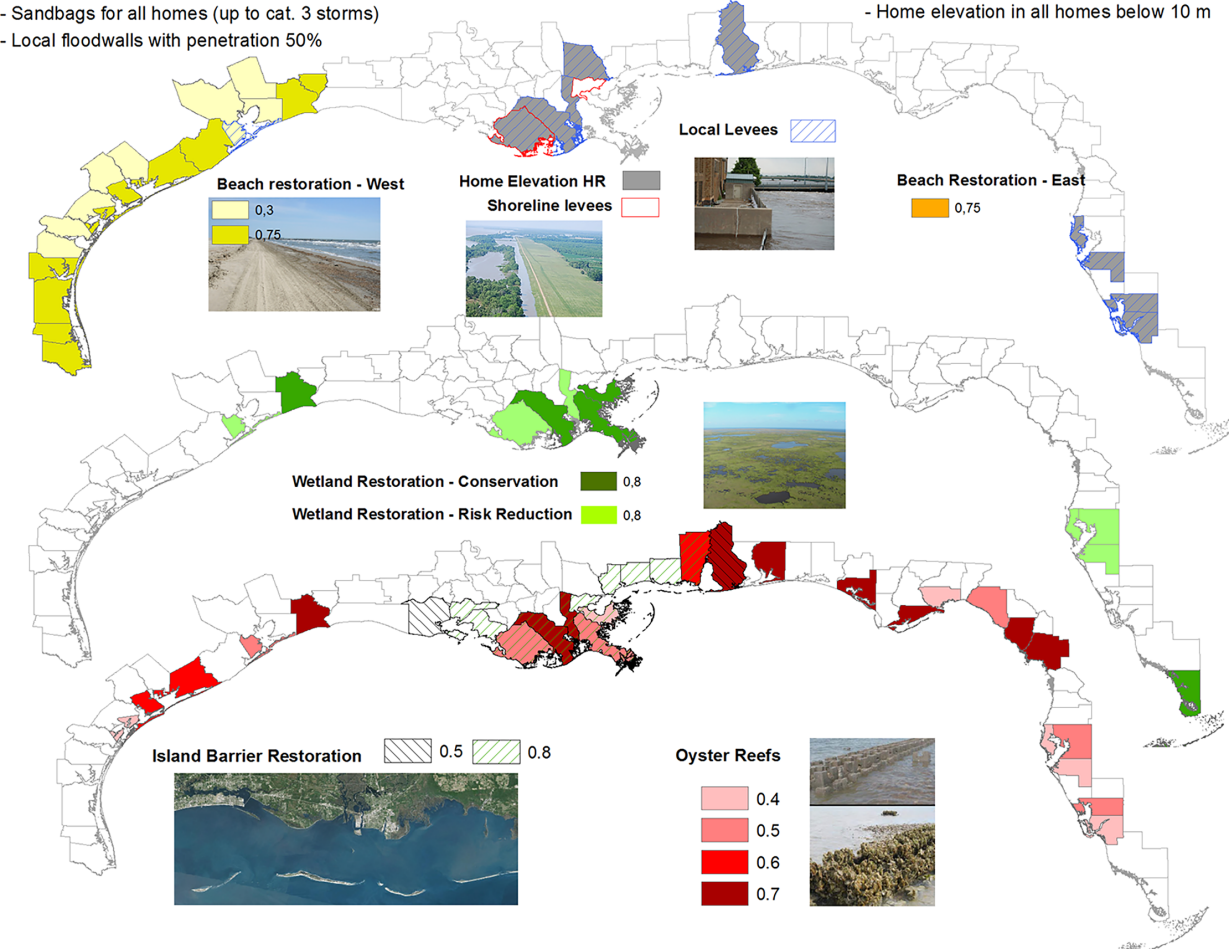
Spatial portfolio of adaptation measures. Adaptation measures are represented by counties where they are implemented. These measures were defined from existing projects and they type of coastline in each county (see Methods). For measures deployed at the shoreline, it is assumed they protect the counties in its lee according to their penetration (for example, sea walls along the shoreline are assumed to protect all the assets in its lee, at each site of implementation). Values and color intensity represent the percentage of assets affected or penetration of each measure (e.g. a penetration value of 0.4 in oyster reefs assumes only 40% of the assets in their lee are protected). It is assumed all assets are protected if no value is given. Sources of images: flickr from U.S. Geological Survey, National Oceanic and Atmospheric Administration, U.S. Fish and Wildlife Service, and U.S. Geological Survey LandSat imagery.

We examine the costs and benefits of ten adaptation measures (outlined in Table 1): (i) green or nature-based measures, i.e. interventions that use ecosystems and natural features to provide hazard attenuation; (ii) artificial or grey measures, i.e. built rigid structures; and (iii) policy measures, i.e. home elevation of high-risk assets. We did not consider adaptation measures that could affect the distribution of assets (e.g. land use policies) or risk transfer (e.g. insurance). Two adaptation measures, wetland restoration and beach restoration, are deployed at different areas of the US Gulf and compared as independent strategies (see Table 1). We discriminate wetland restoration in: (a) high-risk areas: areas at greatest risk, with historical loss of wetlands but where they could be restored; and (b) conservation-priority areas: where most mash area has been lost in the last two decades. Similarly, beach restoration in the western and eastern Gulf is differentiated as two independent adaptation measures. Hereafter, we refer to the ten adaptation measures as the “adaptation portfolio”. The spatial portfolio is mapped in Fig 4 by county. The location of each measure by counties was determined based on existing projects and where they could be feasible in each county, following existing flood guidance in the region [17,82–85]. A review of costs and locations can be found in S3 Table and the detailed locations and length and surfaces of each measure by counties in the Supporting Information.

**Table 1:** Inventory of adaptation measures.

Measure		Description of measure	Locations and implementation	Unitary Cost	Total of units/surface ^a^
Local Levees		6 ft levees built to protect houses	All houses in the counties at highest risk areas	$15,000 /house	1.5 to 1.7 M houses
Sandbags		Sandbags used around homes for all Category 3+ hurricanes	All counties around all houses in low-lying areas (below 10m)	$360/house	2.9 to 3.2 M houses
Dikes		20 ft dikes along the shore	In high risk areas of Houma & New Orleans, LA	$10,000 /ft	340 miles
Home Elevation		Homes elevated by 10 ft	All existing homes in high risk areas	$83,300 / house	1.4 M houses
Wetland Restoration	Risk Reduction Priority	Salt marsh restoration built along shorelines	Wetland restoration in the 6 counties with highest past asset damages & loss of > 25 sq miles of marsh	$25 M /mile	100 miles restored x 0.5ml wide Total: 2.4M acres
	Conservation Priority	Salt marsh restoration built along shorelines	Wetland restoration in the 5 counties that have lost the most marsh in the last 2 decades	$25 M /mile	150 miles restored x 0.5ml wide Total: 3.6M acres
Barrier Island Restoration		Restoration by increasing width	AL & LA counties	$12.1 M/mile	115.5 miles x 1,000 ft
Oyster Reef Restoration		Artificial construction of oyster reefs	Restoration of 25 miles of oyster reefs in each of 24 counties with suitable habitat. For Tampa Bay 25 miles divided among 3 counties	$1.5 M /mile	1,050 miles
Beach restoration	western Gulf (TX)	Artificial beach nourishment to increase width	Coastal beach front across Texas	$22 M /mile	370 miles x 100 ft wide
	eastern Gulf (FL)	Artificial beach nourishment to increase width	All Gulf counties with significant beachfronts across Florida	$22 M /mile	300 miles x 100ft wide

**List of adaptation measures with description of type of measures, summary of locations of implementation, costs and total aggregates, across the Gulf.** The measures assume a 20-year implementation period and include maintenance costs. The costs are discounted at a rate of 2%, with the exception of sandbags and temporary flood-barriers. The ‘risk reduction priority’ areas for wetland restoration are determined from the spatial distribution of risk (S8 Fig), namely: Baldwin (AL), Charlotte (FL), Lee (FL), Manatee (FL), Pinellas (FL), Jefferson (LA), Lafourche (LA), St. Tammany (LA), Terrebonne (LA), Galveston (TX). M–million; ft–feet. A detailed list of cost estimates and references supporting the unitary costs can be found in S3 Table. The measures are distributed in each county considering the shoreline length and the feasibility of implementation in each geography. For this, a review of projects and sources can be found in S3 Table. The total units and surface result from adding the length or surfaces from all counties where each measure is implemented.

^a^ To estimate the total units potentially restored, we include an example for wetland restoration: (1) it is assumed that a restoration wetland unit of 1 mile x 0.5 mile could provide the flood reduction benefits outlined in Table 2; (2) the units of restoration wetlands by counties provides the total length of coastline and total surface of wetland potentially restored (100 miles of 1x0.5 units); (3) the total surface potentially restored is multiplied by the unitary costs (an average value of the revision of unitary costs can be found in S3 Table).

Costs estimates for implementation of the adaptation measures are derived from literature review of restoration and engineering projects in the region [84,86–88]. From them, we estimate average costs that include construction and maintenance and assume fixed dimensions for each measure (e.g. length and width for wetlands, length and height for levees) to calculate a representative cost. Table 1 outlines these unitary costs along with the total cost of each adaptation measure. A more extensive discussion of costs estimates with sources of information can be found in the Supporting Information (S3 Table).

The benefit for each measure is calculated from the potential effectiveness of each measure to: (i) reduce hazards by attenuating waves and surges; (ii) provide physical protection from floods, blocking the water flow (i.e. flood barrier); and/or (iii) avert the physical exposure (e.g. home elevation). These mechanisms were set based on available flood proofing guidance and projects in the region [17,82–85]. Table 2 summaries the effectiveness values used for each measure. The proportion of assets each measure could protect is also factored in, assuming for green measures that not all the assets in the floodplain are protected, but only a proportion of them (40 to 70%, depending on the measure). These values are represented in Fig 2.

**Table 2:** Effectiveness of measures.

Type of Measure	Hazard reduction factor				Elevation Threshold (m)	Mechanism for adaptation implemented in the model
	Wind Waves (%)		Storm Surge (%)			
	Mean	Low	Mean	Low		
Local Levees (homes)	20	20	-	-	1.8	Overtopping (local)
Levees (shoreline protection)	95	95	-	-	7.5	Overtopping (first line of defense or shoreline)
Sandbags (& temporal flood-barriers)	-	-	-	-	0.5	Overtopping (local)
Beach Nourishment (high risk & low risk areas)	75	50	-	-	-	Hazard reduction
Local floodwalls	-	-	-	-	1.2	Overtopping (local)
Home Elevation (new builds and low and high risk areas)	-	-	-	-	3	Elevation of the structure
Wetland restoration (Risk Reduction and Conservation priorities)	60	30	30	15	-	Hazard reduction
Barrier island restoration	60	30	15	5	-	Hazard reduction
Oyster reef restoration	60	30	5	-	-	Hazard reduction

Relation of parameters used for assessing the effectiveness of each measure in reducing the hazards and providing adaptation benefits, for each adaptation measure. The effectiveness of measures is studied based on default or moderate estimates found in the literature (Mean) and a sensitivity test on performances considering more conservative estimates (Low). Only the mean estimates are discussed in the main paper, although the low estimates are used for the sensitivity tests of costs and benefits (see Methods). The estimates were set based on literature review and sources in S2 Table.

To calculate the aggregated benefit of adaptation, we first calculate the total risk over the time period the measures are implemented. For this, we calculate the Annual Expected Damage at the end of the implementation period (e.g. year 2030; Table 3) and discount it to the aggregated Net Present Value (NPV) in the reference year (2010), as indicated previously. We use two discount rates: 2 and 10%, for comparison purposes. When discussing the adaptation results, the NPV of accumulated annual risk is referred as the ‘Total Risk’ in the adaptation period. We also discount analogously the costs and benefits of each adaptation measure to their NPV for each period (20 and 40 years). The total benefit of each measure is compared with the ‘total risk’ in NPV terms. Sensitivity analyses are performed to examine how varying the costs and benefits factors of green measures affects cost effectiveness. The sensitivity scenarios include: (i) one moderate scenario (default) with average estimates of effectiveness and costs for both green, grey and policy measures; (ii) one more conservative scenario with lower estimates of effectiveness for nature-based measures (Table 2), but without changing the costs; and (iii) the most conservative scenario with both increased costs and reduced effectiveness for the green measures. The Results section focuses on the moderate scenario for the sake of simplicity, although the comparison with the other scenarios are briefly discussed when relevant. Detailed results for the other two scenarios are included in the Supporting Information.

**Table 3:** Present and future risk.

Estimate	Present Risk (bill.$)	Year	Future Economic Exposure Growth scenario	Economic Contribution (bill.$)	Climate Contribution (bill.$)	Total Future Risk ^a^ (bill.$)
Annual Expected Damages	4.25	2030	Low	34%	48%	7.72
			High	76%	63%	10.18
		2050	Low	86%	144%	14.04
			High	237%	260%	25.40
1-in-100-yr	87.9	2030	Low	34%	29%	142.90
			High	76%	38%	188.55
		2050	Low	86%	65%	220.59
			High	236%	118%	398.98

Present and future risk, measured in terms of Annual Expected Damages (AED) and loss with probability 1-in-100-yr. Future risk separates the contribution of future economic exposure and climate change. Values are expressed in billion US$. The risk in the future is calculated by combining the future exposure (asset value) with the future climate. The contribution of economic development and climate change is assessed independently by calculating the risk in the future with (see Methods): (i) the future exposure and present climate and (ii) present exposure and future climate. The ‘Low’ economic represents a compound annual growth rate in assets of 1%; while the ‘High’ scenario assumes a 2% rate. Results are organized by years, for the future horizons of 2030 and 2050.

(a) Future risk values correspond to the target year and are not expressed in Net Present Value.

## Results

### Current and future risk under different drivers

**Fig 5:**
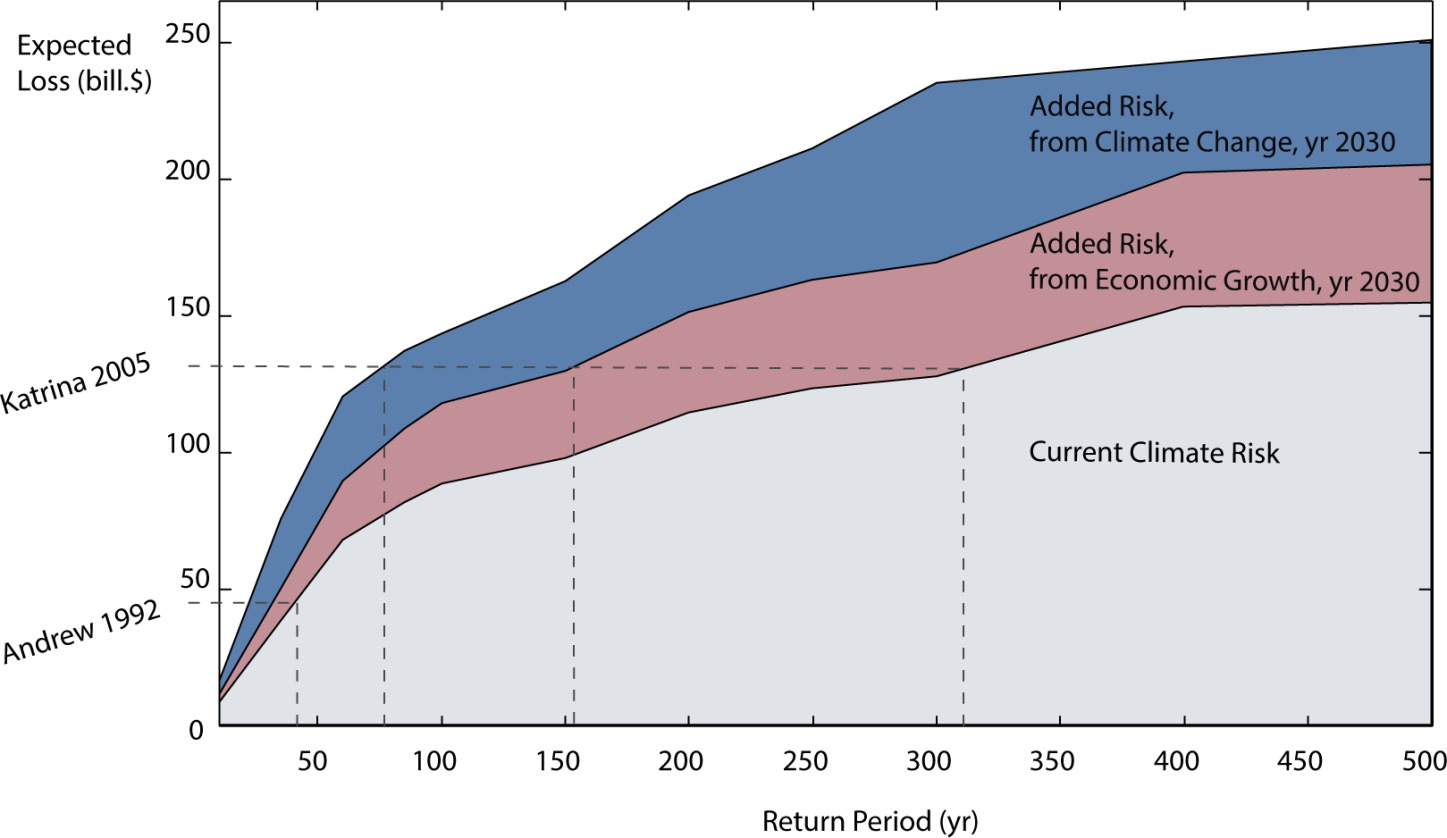
Future damages-frequency curve compared to present. Values represent the expected losses for each return period (a return period R, has a probability of occurring of 1-in-R-years) across the US Gulf Coast. Current Risk is shown in grey. The contributions of the future economic exposure (red) and the change in climate (blue; which includes subsidence, sea level rise and changes in storms) are marked atop current climate risk. The dashed lines show the costs of Katrina and Andrew (updated to 2015 US$’s) for reference purposes. The contribution of economic development and climate change is assessed by calculating the risk in the future with (see Methods): the future exposure and present climate (red), and present exposure and future climate (blue).

Fig 5 represents the risk in a damages-frequency curve, differentiating the contribution of climate change and economic exposure. For comparative purposes, the Figure includes the historical loss from Katrina (2005) and Andrew (1992), adjusting both figures to US dollars in 2015. The damages from Hurricane Katrina in 2005 were more than $131.1 billion in current value (108 billion in 2005 dollar value, according to the National Oceanic and Atmospheric Administration), and corresponds to a frequency of over 1-in-300-yr. Fig 5 shows an equivalent loss will have a higher probability in the future. From economic exposure growth and climate change, an event like Katrina would be below the 1-in-100-yr probability by 2030.

Table 3 provides estimates of present and future risk differentiating the contributions of economic exposure and the climate hazard. The Annual Expected Damage (AED) across the Gulf is currently estimated in $4.25 billion, but could increase significantly from both increased exposure (34–76% increase, for low and high growth scenarios respectively, see Table 3) and climate change (48–63%). For extreme losses, the current 1-in-100-yr flood loss is estimated to be $88 billion (Table 3). However, in 20 years the 1-in-100-yr flood loss will rise from $88 billion to $143–188.5 billion (Table 3) from the joint effect of climate change (29–38%) and economic growth (34–76%). The risk of the 1-in-100-yr flood loss could reach $188.6 billion for the most aggressive economic scenario (Table 3). As the effects of climate change, land subsidence, and higher economic exposure progress in the coastal areas of the Gulf, risk will increase significantly by the year 2050 (Table 3). Overall risk, from the effect of both economic concentration of assets in the coastal zone and climate change, could more than double the risk estimates by 2050 compared to 2030.

Table 3 also shows that economic exposure growth, a major driver of risk in the region in the past, will continue to be a critical factor in the future. Coastal development in the coastal zone is the main driver behind extreme infrequent losses, and will continue to be the main factor of major future disasters. Table 3 shows that the economic contribution to the 1-in-100-yr loss overcomes the contribution of climate (34% economic to 29% from climate for low growth, and 76% to 38% for high growth respectively) and contrasts with the contributions in AED that are more balanced for both drivers (34% economic to 48% from climate, and 76 to 63% respectively, see AED contributions by 2030 in Table 3).

However, climate change will cause larger future annual expected losses. The effect of climate will become dominant in annualized values, as expressed by the Annual Expected Damages. The combined action of storms, sea level rise and land subsidence could raise AED in 48–63% by 2030 and in 144–260% by 2050 with respect to present estimates (Table 3). This effect is amplified as climate changes become more acute into the future when the changes in climate by 2050 will contribute significantly more to AED than the economic driver: 144% compared to 86% (Table 3). However, even in the year 2050 the largest losses, as measured by 1-in-100-yr loss, will still be mostly driven by the growth in the coastal exposure (Table 3).

### The economics of adaptation and risk reduction

**Fig 6:**
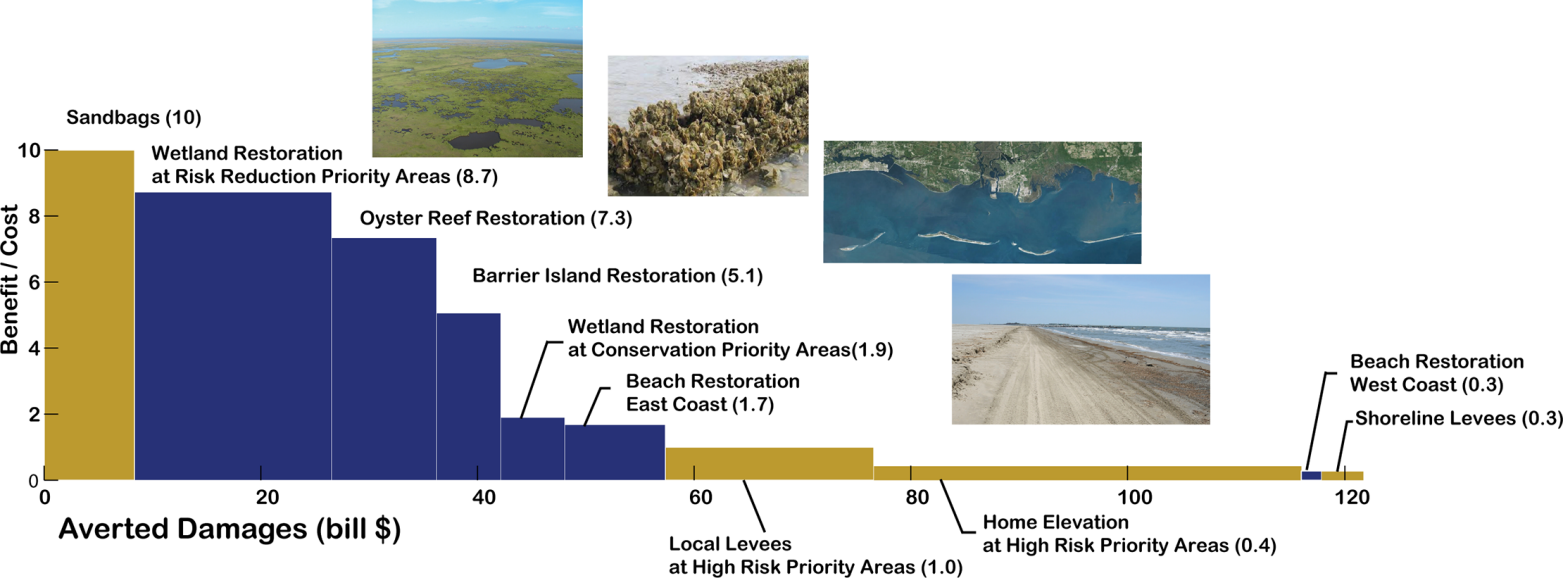
Cost-benefit analysis. Comparison of the costs and benefits of the adaptation measures. Benefit to cost ratios are represented in the vertical axis (height of the bars), with the horizontal axis noting the aggregated benefit (i.e. total averted damage), and the width of the bars the individual benefit from each measure. The blue bars identify nature-based adaptation measures, while the brown color represent the remaining adaptation measures. The values correspond to net present values with a 2% discount rate, for low future economic exposure growth and an implementation period of 20 years. Sources of images: flickr from U.S. Geological Survey, National Oceanic and Atmospheric Administration, U.S. Fish and Wildlife Service, and U.S. Geological Survey LandSat imagery.

Fig 6 shows a cost-benefit analysis on different adaptation measures to assess their relative cost effectiveness for reducing risk. The adaptation portfolio of measures is organized in decreasing order of benefit to cost ratios. The heights of the bars represent the benefit to cost ratios, the width the total benefit for each adaptation measure. Table 4 outlines these ratios and the total benefits for each measure, and includes also results for other scenarios to test sensitivity to costs and effectiveness.

**Table 4:** Benefit to cost ratios and total benefits.

		Moderate estimates		More Conservative estimates (effectiveness)		Most Conservative estimates (effectiveness and costs)	
MEASURE NAME		B/C	TB	B/C	TB	B/C	TB
Local Levees		0.99	19.2	0.99	19.2	0.99	19.2
Sandbags		10.00	8.4	10.00	8.4	10.00	8.4
Dykes & Levees		0.26	3.9	0.26	3.9	0.26	3.9
Home Elevation		0.73	39.4	0.41	39.4	0.41	39.4
Wetland Restoration	Risk Reduction Priority	8.72	18.2	5.03	10.5	4.19	10.5
	Conservation Priority	1.90	5.9	1.00	3.1	0.83	3.1
Barrier Island Restoration		5.07	5.9	1.95	2.3	1.63	2.3
Oyster Reef Restoration		7.34	9.7	2.17	2.9	1.81	2.9
Beach nourishment	western Gulf (TX)	0.28	1.9	0.19	1.3	0.16	1.3
	eastern Gulf (FL)	1.68	9.3	1.15	6.4	0.96	6.4
*Total cost-effective adaptation*		* *	*57*.*4*	* *	*33*.*6*	* *	*24*.*1*
*Nature-based cost-effective adaptation*		* *	*49*	* *	*25*.*2*	* *	*15*.*7*

B/C–Benefit to Cost Ratio

TB–Total Benefit (bill. $)

Benefit to cost ratios and total benefit for the list of adaptation measures, across scenarios of costs and effectiveness. The total climate risk for the scenario is $134 billion (calculated from Annual Expected Damages over a 20-year period). Total cost-effective adaptation is the aggregated value of the total benefits (TB) for all measures with benefit to coast ratio (B/C) above 1. The nature-based cost-effective adaptation is the aggregated value of the total benefits only for the nature-based measures (wetland restoration, beach nourishment, barrier island restoration, and oyster reef restoration) with a benefit to cost ratio above 1. Values correspond to the low economic growth for the year 2030. The discount rate of benefits and costs is 2%. In columns, the ‘more conservative’ and ‘most conservative’ cost-benefit scenarios correspond to (see Methods): the sensitivity analysis where the effectiveness for green measures are reduced (see Table 2) and the costs increased by 20% (costs are only modified in the most conservative scenario). Values for the moderate scenario are represented in Fig 6. A similar representation of other scenarios can be seen in S9 Fig.

Sandbags are a cheap and temporary measure with the highest benefit to cost ratio (bar height), therefore the most cost-effective. However, they offer low overall risk reduction, i.e. benefit (bar width), compared to other measures (Fig 6, Table 4). Nature-based measures, particularly marsh and oyster reef restoration, are among the most cost-effective measures (high benefit to cost ratios) and together contribute the most to overall damage reduction (total benefit) from among the combo of cost-effective risk reduction measures in the analysis (Table 4). Nevertheless, grey and policy measures such as local levees and elevation of homes could deliver the greatest damage reduction benefits for a single measure, but they are expensive to implement over large areas and are not cost-effective overall, i.e. benefit to cost ratio below 1 in Fig 6.

For wetland and beach restoration, we compare the same adaptation measures in distinct locations of the Gulf (see Table 1 for description and costs, and spatial distribution in Fig 4): (i) wetland restoration in high risk areas versus conservation priority areas; and (ii) beach nourishment in the eastern and western Gulf. The results, shown in Table 4, confirm very different benefit to cost ratios between them demonstrating that location matters for risk reduction. Although wetland restoration results cost-effective in both locations, restoration in high-risk areas is over 4 times more cost-effective than in priority areas for conservation (benefit to cost ratios of 8.7 versus 1.9, Table 4). For beach restoration, only in the eastern Gulf it results cost-effective and by 6 times more than in the western margin (1.68 versus 0.28, Table 4). The risk reduction benefit of these measures is higher than their counterparts because they are implemented in areas more exposed to hurricanes and with higher asset values, as seen in the Benefit column in Table 4. Although changes in the cost and performance assumptions result in different benefit to cost ratios, green measures are found consistently with high benefit to cost ratios in a sensitivity analysis (see Methods) and with values above 1, thereby resulting cost-effective options across the scenarios analyzed. Results of sensitivity analyses in the cost and benefit factors are shown in Table 4 and represented in S8 Fig.

With Annual Expected Damage of $7.72 billion by 2030 (Table 3), the total risk over a 20-year time period is estimated to total $134 billion (net present value of all the annual expected damages for a low economic growth scenario up to year 2030, see Methods). The most cost-effective adaptation combo (measures with benefit to cost ratios over 1 in Table 4) could avert approximately $57.4 billion in damages. This represents a 42.8% reduction of the total risk. The majority (85%) of the cost-effective risk reduction could be averted by nature-based measures, for a total of $49 billion. Even under conservative estimates, 65% of nature-based adaptation is cost-effective (15.7 over 24.1 billion, Table 4).

This is also true for more aggressive economic exposure growth. For comparison, under a high economic growth scenario, the aggregated annual risk over a 20-year period would total $176.6 billion. The implementation of cost-effective measures could avert $101 billion in damages (57.2% of the total risk), of which $64.6 billion could come from nature-based measures (S4 Table).

By 2050, the cost effectiveness of adaptation is even more attractive. The total risk will vary significantly depending on the future economic growth, from $398–720 billion (low and high economic growth, over 40 years). Most of the adaptation portfolio becomes cost-effective (including the large systems of floodwalls and levees) and, nature-based adaptation in the portfolio would represent approximately 47.8% (S5 Table).

## Discussion

The need for adaptation and risk reduction is growing. This work demonstrates that it is possible to build a quantitative economic case for adaptation measures that include nature-based solutions and at scales relevant for planning. First, our assessment of damages helps to discriminate how much each driver of risk contributes to the future risk. While climate change will be responsible for more frequent, relative small losses, a higher economic exposure in the coastal zone (i.e. from aggressive coastal development) will continue to be the greatest driver of coastal risk. This conclusion has important implications for both coastal management and risk reduction strategies because land use and coastal management policy have direct influence over development in the coastal zone.

The results show that some adaptation measures can be particularly cost-effective in addressing the risking risks. We were able to compare cost-effectiveness of a number of adaptation measures, including green and grey options and find that a suite of cost-effective options could avert these future damages considerably. Cost-effective options to reduce risks (i.e. measures with benefit to cost ratio above 1) includes a combination of measures (green and grey), which could potentially avert up to 42.8–57.2% of total climate risk ($57.4–101 billion in damages) over the next 20 years (Table 4). Nature-based adaptation, in particular, could be among the most cost-effective options. The nature-based measures in the analyzed portfolio may help to avert 36.6% of total climate risk ($49–64.6 billion), with an average benefit to cost ratio of 3.7 to 4.9 (at an aggregate cost of $13.2 billion; Table 4). As sea level rises, land subsides, storms increase in frequency and intensity, and assets in the coastal zone increase, all adaptation measures become more cost-effective over time. This also has practical implications for coastal management, as the benefits of any funds that managers are able to spend now to reduce risks will compound with time.

Some measures such as home elevation can provide the most protection, but they are expensive to implement and thus have a low benefit to cost ratio (Table 4). This result suggests that, while cost-effectiveness is an important consideration, adaptation decisions should also take into account overall protection (or general effectiveness). Furthermore, cost effectiveness and general effectiveness may be two of many factors for adaptation planning. Other considerations, such as the social benefits and vulnerability, environmental benefits, allowable regulations, perceived risk and other social perceptions are also in play.

Assessing how much it is worth now to prevent future damages is not a simple issue. Discounting plays a significant role in assessing cost effectiveness when there are significant differences in the timing of costs (now) and benefits (future). Several factors, including interest rates and perceived costs, influence how adaptation is discounted to present dollar-value. There is vigorous debate on the appropriate discount rate for adaptation benefits [89–91]. Lower discount rates (2%), associated with social discounting (rates used in computing the value of funds spent on social projects), provide higher benefit to cost ratios, making investments in protection today more attractive, while more aggressive discounting rates (10%) make it easier to pay for these risks and damages in the future (but delay their implementation now). Here, we used both values as a way to measure the range of variation in benefit to cost ratios. While changes in the discount rate affect which measures result currently cost-effective (benefit to cost ratios below 1, see S6 Table), they do not change the ordering of which measures are most cost-effective.

This analysis may also stimulate a rethinking of the places where restoration is prioritized. Conservation outcomes are principally achieved through the protection of intact habitat or the restoration of degraded habitat. In general, restoration is generally considered a lower priority action than protection, but recent research shows that both are crucial components of a conservation strategy to optimize biodiversity or ecosystem services, and in some circumstances, restoration should be preferred [92]. The geographic location of adaptation measures influences their cost effectiveness and general effectiveness, which was particularly true for beach and wetland restoration. When nature-based measures are implemented in areas with higher asset value and at greater risk, their cost effectiveness increases significantly. Habitat conservation and restoration actions are often prioritized in areas with lower population and development (e.g., Texas as compared to Florida in beach restoration, and South-East Louisiana for marsh restoration). However, this study shows that it would be important to restore habitats not only in places with conservation values but also risk reduction benefits, helping to meet non-exclusive multiple goals.

The actual costs for each measure (e.g. marsh restoration) can vary strongly across the US Gulf and will depend on many local factors too. Here, we use representative average values and apply them homogenously throughout the region as a first cut. The costs of some measures, such as oyster reef restoration, are likely to be fairly consistent [93–95] (see review of costs in S3 Table). However, in cases where land acquisition or permits are required (e.g. for marsh restoration in high-value areas with strong market forces at play), the cost may vary significantly. We consider several scenarios as a way to manage this uncertainty, but this is one area where future research, specifically downscaling and more granularity, will be particularly useful.

Our analyses and results also highlight key areas for further research. For example, while there are studies that provide a more detailed coastal hazards description in the region [69,70,96], it is important to advance in combining hazard information with economic damages and adaptation assessments. This is, however, challenging. The use of complex numerical modeling is usually restricted to smaller areas and to a limited number of scenarios. These limitations could be addressed through hybrid modeling approaches that combine numerical modeling, statistical analysis and downscaling techniques. Furthermore, this study shows that future economic exposure in the coastal zone can be a major driver of risk. Indeed, exposure to hazards is influenced by land use and coastal policy, but research rarely considers how future coastal development could influence future risk and the design of adaptation strategies. More detailed adaptation scenarios should also factor in local characteristics, spatial variations and local economic exposure, but at scales relevant for informing planning.

This study assumes that all measures are static and does not account for the fact that ecosystems can adapt and grow with changing environmental conditions, such as sea level rise, which would add to their cost effectiveness relative to built infrastructure. For example, wetland accretion can help building land with a rising sea [36] and healthy oyster reefs have also been shown to be able to keep up with sea level rise [34]. In contrast, built structures cannot self-adapt and would require upgrade planning. These considerations were not part of the analysis.

Nature-based measures also offer other environmental and ecosystems service beyond coastal protection that would further increase their benefit to cost ratio. For example, restored ecosystems can offer important tourism and recreational services, including recreation, fishing and hunting as well as cultural services that may also play a vital role in identifying and prioritizing restoration and conservation priorities from a socio-economic perspective [97]. Beaches, for example, provide important coastal protection benefits, but they also attract travel and tourism, both major industries and employers, making them critical to local, state and the national economies [98]. Hence, it is important to manage coastal ecosystems to reduce stressors so they can be healthy and more resilient to sea level rise and climate change to provide both risk reduction and environmental benefits.

To build coastal resiliency, it is important to promote resilient infrastructure and solutions able to reconcile multiple goals. A multi-objective perspective can also generate funding sources and opportunities for environmental restoration, adaptation and risk reduction. As an example, the RESTORE Act in the US Gulf established the ‘Gulf Coast Restoration Trust Fund’ in response to the Deepwater Horizon oil spill, to restore and protect the natural resources and ecosystems [99]. Assessing the risk reduction potential of restoration projects could support and inform funding allocation from the RESTORE Act into restoration that also would reduce risks to people. Similarly, insurance incentives can help create opportunities to meet goals in risk reduction and support environmental conservation as an added benefit.

## Supporting information

S1 File Supplementary methods and results: ‘Supplementary_methods_and_results.doc’. (DOC)

S2 File Permission and sources of photos. ‘Permission and sources of photos.doc’.
(DOCX)

S1 Fig Statistics of simulated storms based on historical tracks. The simulation reproduces the statistical distribution of Accumulated Cyclone Energy (upper left panel), the number of tropical storms (upper right), total number of hurricanes (lower left) and major hurricanes, i.e. category 3 or above (lower right).
(PNG)

S2 Fig Wave height validation. Significant wave height at NOAA buoy 42040 (29.212 North, 88.207 West, 164.6 m deep).
(TIF)

S3 Fig Relative sea level rise. Historical relative Sea Level Rise trends (mm/yr) from NOAA [75] and the subsidence field digitalized from Ivins et al [77].
(TIF)

S4 Fig Study units and hazard centroids. The centroids (red dots) are the basic units where hazards are calculated and then associated to census tracts.
(PNG)

S5 Fig Value of assets in the US Gulf. Spatial distribution of value of assets in low-lying zones across the Gulf (below 10 m ground elevation) by county level. The topographic distribution of asset value aggregates for the Gulf is plotted in a subpanel (bar graphic), where the x-axis represents ground height and the y-axis total asset value across the Gulf.
(PNG)

S6 Fig Adaptation model. **Representation of the model to estimate adaptation for each measure**. The S-shape curves represent how adaptation is considered in the damage curves: Hazard Reduction; first line of defense until overtopped (FL); local overtopping (OV); and elevation of structures (EL). MSL: Mean Sea Level, FH: Flooding Height onshore; Zi: topographic elevation at each site (i.e. aggregated at study units).
(TIF)

S7 Fig Risk now and in the year 2030. Risk evolution between 2010 and 2030 for the two economic scenarios: Low economic exposure growth (left panels), and high economic exposure Growth (right panels). Bars represent current risk (left) and future risk (right), and separate between the contribution from future economic exposure and climate. Upper panels (a,b) represent the Annual Expected Damage, while the lower panels (c,d) represent the 1-in-100-yr risk.
(TIF)

S8 Fig Annual expected damage by census tract across the US Gulf. Upper panel shows Annual Expected Damage (AED) by county, with reference in year 2010. Lower panel shows the change in AED in 2030, showing areas with the greatest added risks from low economic exposure growth and climate change. Values are given in US$ Millions.
(TIF)

S9 Fig Comparison of the costs and benefits of adaptation measures. Benefit to cost ratios are represented in the vertical axis (height of the bars), with the horizontal axis noting the aggregated benefit (i.e. total averted damage), and the width of the bars the individual benefit from each measure. Panel a: default estimates of protection and cost, equivalent to Fig 6; Panel b: reduced hazard reduction potential by green options; Panel c: both reduced hazard reduction and increased cost for green options. The scenario corresponds to low discount (2%) and assumes a low economic exposure growth in the next 20 years.
(TIF)

S1 Table Present and future exposure in the Gulf. Total asset value at reference date (2010) and two future timeframes (2030 and 2050), for the two scenarios of economic exposure growth.
(DOCX)

S2 Table Hazard attenuation from different coastal features. Range of parameters found in the literature and data sources for attenuation of hazard for different coastal features, along with a brief description of the basic principle for protection.
(DOCX)

S3 Table Cost estimates and sources of information. Review of cost estimates and sources of information for the definition of the adaptation measures.
(DOCX)

S4 Table Benefit to cost ratios for year 2030 under a high economic exposure growth. Benefit to cost ratios and total benefit for the list of adaptation measures, across scenarios of costs and effectiveness. The total climate risk for the scenario is 176.6 US$ billion (calculated from Annual Expected Damages over a 20-year period). Total cost-effective adaptation is the aggregated value of the total benefits (TB) for all measures with benefit to coast ratio (B/C) above 1. The nature-based cost-effective adaptation is the aggregated value of the total benefits only for the nature-based measures (wetland restoration, beach nourishment, barrier island restoration, and oyster reef restoration) with a benefit to cost ratio above 1. Values correspond to the high economic exposure growth for the year 2030. The discount rate of benefits and costs is 2%. In columns, the ‘more conservative’ and ‘most conservative’ cost-benefit scenarios correspond to (see Methods): the sensitivity analysis where the effectiveness of green measures are reduced and the costs increased by 20% (costs are only modified in the most conservative scenario).
(DOCX)

S5 Table Benefit to cost ratios for year 2050 under a low economic exposure growth. Benefit to cost ratios and total benefit for the list of adaptation measures, across scenarios of costs and effectiveness. The total climate risk for the scenario is 398.2 US$ billion (calculated from Annual Expected Damages over a 40-year period). Total cost-effective adaptation is the aggregated value of the total benefits (TB) for all measures with benefit to coast ratio (B/C) above 1. The nature-based cost-effective adaptation is the aggregated value of the total benefits only for the nature-based measures (wetland restoration, beach nourishment, barrier island restoration, and oyster reef restoration) with a benefit to cost ratio above 1. Values correspond to the low economic growth for the year 2050. The discount rate of benefits and costs is 2%. In columns, the ‘more conservative’ and ‘most conservative’ cost-benefit scenarios correspond to (see Methods): the sensitivity analysis where the effectiveness of green measures are reduced and the costs increased by 20% (costs are only modified in the most conservative scenario).
(DOCX)

S6 Table Effect of discounting rates. Benefit to cost ratios and total benefit for the list of adaptation measures, across scenarios of costs and effectiveness. Total cost-effective adaptation is the aggregated value of the total benefits (TB) for all measures with benefit to coast ratio (B/C) above 1. The nature-based cost-effective adaptation is the aggregated value of the total benefits only for the nature-based measures (wetland restoration, beach nourishment, barrier island restoration, and oyster reef restoration) with a benefit to cost ratio above 1. Values correspond to the low economic growth for the year 2030. The cost-benefit scenario corresponds to the default setting.
(DOCX)
