# Supplementary material for: Comparing the cost effectiveness of nature-based and coastal adaptation: A case study from the Gulf Coast of the United States
Source: PLoS One. 2018 Apr 11;13(4):e0192132. doi: 10.1371/journal.pone.0192132 (PMC5894966; doi:10.1371/journal.pone.0192132)
Supplement: S1 File — (DOC) [file pone.0192132.s001.doc]

**SUPPLEMENTARY INFORMATION**

1. **Additional information on hazard definition**

S1 Fig represents different characteristics of the storm simulation and the comparison with the historical set.

To define the pressure field of each tropical storm, we use the Hydromet-Rankin Vortex model [1]:

$P_{r}=P_{0}+\left(P_{N}-P_{0}\right)\cdot e^{\left(-\frac{R}{r}\right)}$ (1)

where P_0_ is the pressure at the center of the hurricane (mb), P_r_ is the pressure at a distance r from the center (km), P_N_ is the pressure outside the hurricane (usually1013 mb), and R is the radius of the maximum cyclostrophic winds (km), with $R=0.4785\cdot P_{0}-413.01$. These parameters can be obtained from the historical storms records and the climada storm simulation routines - see [2].

From the information of the storm tracks, the model (see CLIMADA coastal hazards module[[1]](#footnote-2)) infers the spatial field of pressure and winds, and from them the effect on waves and surges. These models are explained below.

For the wind field, the model uses a non-symmetric fields model [3], where the maximum gradient of winds *U_R_* (km/h) can be obtained from:

$U_{R}=21.8\cdot\sqrt{P_{N}-P_{0}}-0.5\cdot f\cdot R$ (2)

Where *f* is the Coriolis parameter$f=2\omega\sin\left(\varphi\right)$ and $\omega=0.2618rad/h$y $\varphi$ is the latitude in degrees

The wind velocity (10 m; km/h) at a distance r from the center of the storm is: $W=0.886\left(F_{V}\cdot U_{R}+0.5\cdot V_{F}\cos\left(\theta+\beta\right)\right)$ (3)

With $\theta+\beta$ being the angle between the hurricane moving speed $V_{F}$ and the wind speed at distance R from its center, *U_R_*. $F_{V}$ is a damping factor that depends on the relative location to the storm:

$F_{V}=1-0.971\cdot\exp\left(-6.826\left(\frac{r}{R}\right)^{4.798}\right)$ for $\frac{r}{R}<1$ (inside the eye of the storm) , and

$F_{V}=\exp\left(A\cdot\ln^{3}\left(\frac{r}{R}\right)\cdot\exp\left(B\cdot\ln\left(\frac{r}{R}\right)\right)\right)$ for $\frac{r}{R}\geq1$ (outside the eye)

A and B are obtained as:

$A=-0.99\cdot\left(1.066-\exp\left(-1.936\cdot\left(\frac{f\cdot R}{U_{R}}\right)\right)\right)$ and $B=-0.357\cdot\left(1.4456-\exp\left(-5.2388\cdot\left(\frac{f\cdot R}{U_{R}}\right)\right)\right)$

We then calculate wind waves generated by the wind field using three different models, for comparison purposes [3–5]:

- Bretschneider et al (1990) [3], for Hs from a non-stationary cyclone at offshore depths

$Hs=0.2557\cdot F_{h}\left(1-\frac{6.69\cdot N_{C}}{1+10.3\cdot N_{C}-3.25\cdot N_{C}^{2}}\right)\cdot\sqrt{R\cdot\left(P_{N}-P_{0}\right)}\cdot\left(1+\frac{V_{F}\cdot\cos\left(\theta+\beta\right)}{2\cdot U_{R}\cdot F_{V}}\right)^{2}$ (4)

$$Ts=12.1\sqrt{\frac{Hs}{g}}$$

- Young (1988) [4], adjusting and calibrating the Hs max with numerical modeling at offshore depths.

$\frac{g\cdot H_{S}^{\max}}{V_{\max}^{2}}=0.0016\left(\frac{g\cdot x}{V_{\max}^{2}}\right)^{0.5}$ (5)

$$\frac{g\cdot Tp^{\max}}{2\pi\cdot V_{\max}}=0.045\left(\frac{g\cdot x}{V_{\max}^{2}}\right)^{0.33}$$

- Shore Protection Manual [5], modified to include some of the near-shore depth induced effects on the waves (Hs and Tp)

$\frac{g\cdot Hs}{U_{A}^{2}}=0.25\cdot\tanh\left[0.6\left(\frac{g\cdot h}{U_{A}^{2}}\right)^{0.75}\right]\cdot\tanh^{0.5}\left\{\frac{4.3x10^{-5}\left(\frac{g\cdot2F}{U_{A}^{2}}\right)}{\tanh^{2}\left[0.6\left(\frac{g\cdot h}{U_{A}^{2}}\right)^{0.75}\right]}\right\}$ (6)

$$\frac{g\cdot Tp}{U_{A}}=8.3\cdot\tanh\left[0.76\left(\frac{g\cdot h}{U_{A}^{2}}\right)^{0.375}\right]\cdot\tanh^{1/3}\left\{\frac{4.1x10^{-5}\left(\frac{g\cdot2F}{U_{A}^{2}}\right)}{\tanh^{3}\left[0.76\left(\frac{g\cdot h}{U_{A}^{2}}\right)^{0.375}\right]}\right\}$$

S2 Fig shows a comparison of the three wave models at a buoy in the Gulf.

‘Storm Surge’ refers to the water height above predicted astronomical tide level due to the inverse barometer effect and the wind stress over the sea surface [6]. Storm Surge is supposed to be the linear sum of two effects: (1) elevation due to the atmospheric pressure and (2) the wind shear stress over the sea surface. The storm surge sea level pressure effect spatial variation is supposed stationary and symmetric, only depending on the pressure gradient, and is calculated as [7]**:**

$\eta_{vp} =\frac{\left(p_{n}-p_{o}\right)}{\rho g}\left(1-e^{-\frac{\left(R-r\right)}{r}}\right)$ (7)

where $\eta_{vp}$ sea level elevation; P_n_ pressure outside the storm (mbar); P_0_ central pressure (mbar); $\rho$ water density (1025 kg/m³) and R is the hurricane radius (km)

The shear stress produced by the wind on the sea surface generates an elevation of the water level at the coastline, known as Storm Surge. Although its modeling is complex [8,9], the long wave equations can be used to described it on the continental shelf or a lagoon [7]**:**

The sea level surface induced by the wind stress ($\eta_{w}$; SS) can be obtained from the following equation:

$\frac{\partial\eta_{w}}{\partial x}=\frac{n\cdot\tau_{zx}\left(\eta_{w}\right)}{\rho\cdot g\left(h+\eta_{w}\right)}$ (8)

where h is the depth, n is a factor taken that varies between 1.15 and 1.30, and $\tau_{W}$is the wind shear stress in the cross-shore direction.

The wind stress over the water surface is [5]:

$\tau_{W}=\rho\cdot k\cdot U_{\max}\left|U_{\max}\right|$ (9)

where $\rho$ is the water density in kg/m³; $U_{\max}$ is the maximum wind speed (10 m over sea level), m/s; $k$ is a friction factor, O(10^6^) , taken from [10]:

$k=1.2x10^{-6}$ for $\left|U_{\max}\right|\leq5.6$ and $k=1.2x10^{-6}+2.25x10^{-6}\left(1-\frac{5.6}{\left|U_{\max}\right|}\right)$ for

The wind stress acting on the transect is $\tau_{wx}=\left|\tau_{w}\right|\cos\theta$. Although the wind shear stress is usually very small, when its effect is integrated over a large body of water, like the coastal shelf, it results in significant water levels onshore.

The approach assumes an orthogonal transect to the bathymetry and responds to an approximation of the long-wave shoaling on the continental shelf. Conditions are also assumed stationary for each storm position and the maximum wind speed acting on the coastal transect. The relative angle between the maximum wind speed and the bathymetric transect is considered to define the tangential stress and is taken at the beginning of the continental shelf (i.e. where shoaling starts having an effect), here assumed at 200 m deep. Bottom friction and 2D effects are neglected in this approach.

Although there are significant simplifications behind parametrical models like the ones used in this analysis, they are easy to implement and computationally efficient to run in large domains [11]. These characteristics make them suitable for comparison of many different factors and scenarios in a large domain, while conserving the stochastic approach.

The analysis was performed using the ‘‘*CLIMADA’* open source model [2,12], simulating a set of synthetic 15,000 storms from the historical record, for the present (reference in year 2010) and future climates in years 2030 and 2050. The hazard scenarios included the effects of climate change in storms, through changes in intensity and frequency, according to [13] and [14], as well as relative sea level rise:

- We considered the intensity of all categories of storms increased by 11% by the end of the century and the category 4 and 5 hurricanes become an 80% more frequent, while the lower intensity storms become 28% less frequent.
- For future sea levels, we accounted for land subsidence, particularly important in the highly subsiding Mississippi delta [15],
- For sea level rise, historical trends [16] are extrapolated to the target years, which can be considered a conservative approach (S3 Fig).

1. **Economic Exposure**

We extracted asset values from the HAZUS database at a block level, aggregated them by tracts and integrated all the information into a total of 3,238 centroids (S4 Fig). We also processed the assets by ground heights across the Gulf using the national Elevation Dataset [17]. S5 Fig shows the spatial distribution of assets in low-lying areas (i.e. below 10 m of elevation). The assets were subsequently aggregated into centroids (S4 Fig), which constitute the basic units of study and where we calculate damages from the hazards.

The hazard term is not only the unique factor that could change risk in the future, exposure and coastal development also changes with time. Moreover, change in exposure is particularly acute in coastal areas. For example, the Gulf Coast population has increased by 109% since 1970, compared to a 52% increase in the US total [18,19].

We examined future exposure for two economic scenarios of coastal development, for the years 2030 and 2050, based on past and expected growth in the region [18–20]: ‘Low economic exposure growth’ assumes growth in existing assets/year of 1% in value and ‘High economic exposure growth’ assuming a 2 %/year increase.

S1 Table outlines the regional aggregates for the two growth scenarios considered. We updated present asset values and number of houses to the two future timeframes considering: (1) past historical data on the House Price Index by state (ranging from 21.1% in Mississippi to 43.7% in Louisiana, with an average of 2.67% from 2001-2010); (2) historical evolution of economic growth, and (3) projections from the World Bank and PwC Economics [20] for the years 2030 and 2050 (correspond to annual rates of 2.89% and 3.89%, respectively).

1. **Correction of total water levels by landscape features**

Because large coastal geomorphology features (e.g. the presence of embayments, extensive wetlands, barrier islands, and large scale dyke systems like in New Orleans) influence waves and thus flood height, we correct the total water levels when these features are in the pathway of the storm with estimates based on a literature review (see S2 Table). The correction factors consider the reduction potential on both short (wind-waves) and long waves (storm surges). This approach is similar to how we also model adaptation.

For managing uncertainty, we compare damages in three different scenarios: the first scenario assumes the landscape features have no effect in reducing waves (no-defense scenario); the second considers a conservative measure of the effectiveness of these features (high-defense); and the third scenario uses the most likely estimates of the effectiveness (low-defense) of these measures. The variation between the no- and high-defense parameterization is a proxy of present protection offered by the coastal landscape in the Gulf. We compared the risk values with private losses estimates and determined the intermediate parameterization (low defense) was the best representation of losses.

1. **Adaptation and cost-benefit analysis**

We estimate a representative unit cost for each of the measures after literature review of restoration and engineering projects in the region. S3 Table provides the list of estimates from the literature review and the sources of information.

The model also considers that only a percent of study units would benefit from protection of measures, which is referred to as “penetration” in the model [2,12] (i.e. fraction of assets and at what geographies the influence of measures is likely to penetrate). With costs and benefit calculations, the benefit to cost ratios for averted damages from each adaptation measure can be obtained for present and future assets and climate. Total Net Present Values (NPV) of the costs and benefits are obtained after discounting over each timeline. We considered two discount rates: one of 2% and a larger discount rate of 10% to account for a more aggressive discount valuation. Finally, the net present value of the costs of each measure considers 20-year implementation periods and periodic maintenance. See Tables in the main text.

Benefits were calculated assuming three modes of adaptation, summarized in the S6 Fig.

The annualized NPV is the average yearly net return over the lifetime of the suite of adaptation options, that is, the annualized cash flow. The benefit to cost ratio is the ratio of the present value of discounted benefits to the present value of the discounted costs for each of the measures. This outcome of the analysis can provide an intuitive answer regarding the desirability of the measure. If the ratio is greater than one then the project is cost-efficient, and the ratio itself gives the return of investment through the period considered (benefits of reduced losses per dollar spent on the measure). The resulting benefit-cost ratios can be represented in a benefit to cost curve, where the width of each bar along the x axis represents the cumulative potential of that measure to reduce total expected damage up to a target year for a given scenario, and the height of each bar represents the ratio between benefits and costs for that measure. The assembled cost curve shows (from left to right) the range of measures from least cost-efficient to most cost-efficient. The results should thus be used to start discussions on the different measures and the opportunity to avert expected damage, rather than be read as recommendations to implement certain measures.

1. **Supplementary results of risk and drivers**

S8 Fig shows Annual Expected Damage by census tract across the US Gulf.

S9 Fig shows the benefit to cost ratios for the three adaptation scenarios. Only the first panel is shown in the main paper (Fig 6).

S4 Table outlines the benefit-cost ratios for a High Economic growth, for comparison with the Low Economy case (in the main paper). A higher exposure involves larger total benefits from risk reduction measures and more favorable benefit-cost ratios across all scenarios. Note also that as the time period considered for calculating the benefits increases (for example for the climate and the exposure in 2050) these ratios become much more favorable (results for year 2050 in a low growth scenario are outlined in S5 Table).

Finally, for the purpose of comparing discounting rates, S6 Table shows the different benefit-cost ratios obtained for the two different discounting options.

1. **Extended Discussion**

**a) A word on managing uncertainty by comparing scenarios and parameterizations**

Valuation and sensitivity analysis can range from simple presentations of possible ranges of values or confidence intervals to simulation methods exploring the implications of uncertainty in the parameters. This study opted to compare range of values to assess the key parameters and the levels at which the conclusions may change, for example by exploring if the benefit to cost ratio fall below one under a wide set of factors. By changing these parameters across scenarios, we can manage the intrinsic uncertainty and assess what creates the most variation in risk and, more importantly, in the cost effectiveness of risk reduction measures. These results can help to identify areas in which further research to resolve key uncertainties would be justified.

**b) A word on tools and the problem of scales**

The need for better strategic visions for risk reduction are clear; these visions must be guided by national coastal risk assessments that identify areas most at risk and help to prioritize future investments [e.g. ,21]. Cost-benefit analysis and risk assessments are increasingly being used in the context of climate adaptation at local sites [22–24]. However, large-scale assessments [e.g. ,25,26] are mostly useful for global and large regional analyses (i.e. order of hundreds of kms), but they fail to include a probabilistic assessment of hazards and the range of adaptation measures is limited, although they include dynamic interactions of climate change scenarios and socio-economic pathways. Conversely, high-resolution public studies, e.g. Coastal Master Plan in Louisiana [27–29] and public risk models, e.g. HAZUS – Flood [30,31], work at much higher resolutions (resolutions of 100 m) but are only applicable to small areas. Furthermore, all of them they rarely include natural ecosystems. There is a need of tools at a regional scale bridging the gap between large-scale assessments and high-resolution studies that also account for the natural protection that ecosystems offer and to assess the benefits and costs of green infrastructure for coastal defense.

Recently, [32] following the method in [33], provided US-wide flood risk estimates as well as cost of nourishment, armoring and managed retreat as adaptation options for different climate scenarios. Our application in the US Gulf further shows how these set of tools and approaches are becoming increasingly available.

**Supplementary References**

1. Holland GJ. An Analytic Model of the Wind and Pressure Profiles in Hurricanes. Mon Weather Rev. American Meteorological Society; 1980;108: 1212–1218. doi:10.1175/1520-0493(1980)108<1212:AAMOTW>2.0.CO;2

2. Bresch DN. Climada the open source NatCat model; model code, tropical cyclone and storm surge module, and documentary material [Internet]. 2014. Available: https://github.com/davidnbresch/climada

3. Bretschneider CL. Tropical cyclones. In: Herbich JB, Bretschneider CL, editors. Handbook of Coastal & Ocean Engineering. Houston: Gulf Pub. Co.; 1990. pp. 249–303.

4. Young I. Parametric Hurricane Wave Prediction Model. J Waterw Port, Coastal, Ocean Eng. American Society of Civil Engineers; 1988;114: 637–652. doi:10.1061/(ASCE)0733-950X(1988)114:5(637)

5. USACE. Shore Protection Manual [Internet]. US Army Corps of Engineers; 1984. Available: http://scholar.google.com/scholar?hl=en&btnG=Search&q=intitle:Shore+Protection+Manual#0

6. Losada IJ, Reguero BG, Méndez FJ, Castanedo S, Abascal AJ, Mínguez R. Long-term changes in sea-level components in Latin America and the Caribbean. Glob Planet Change. 2013;104: 34–50. doi:10.1016/j.gloplacha.2013.02.006

7. Dean RG, Dalrymple R a. Water Wave Mechanics for Engineers and Scientists. Liu PLF, editor. World Scientific; 1991.

8. Resio DT, Westerink JJ. Modeling the physics of storm surges. Phys Today. 2008;61: 33–38. doi:10.1063/1.2982120

9. Jacobsen B. Hurricane Surge Hazard Analysis : The State of the Practice and Recent Applications for Southeast Louisiana. 2013; 534.

10. Van Dorn W. Wind Stress on an artificial pond. J Mar Res. 1953;12.

11. Silva R, Govaere G, Salles P, Bautista G, Diaz G. Oceanographic vulnerability to hurricanes on the Mexican coast. Proc 28th International Conference on Coastal Engineering. Singapure: World Scientific; 2002.

12. Bresch DN, Mueller L. Climada Manual. 2014; 1–73.

13. IPCC. Managing the Risks of Extreme Events and Disasters to Advance Climate Change Adaptation [Internet]. Field CB, Barros V, Stocker TF, Dahe Q, editors. A Special Report of Working Groups I and II of the Intergovernmental Panel on Climate Change. Cambridge, UK, and New York, NY, USA: Cambridge University Press; 2012. doi:10.1017/CBO9781139177245

14. Walsh KJE, McBride JL, Klotzbach PJ, Balachandran S, Camargo SJ, Holland G, et al. Tropical cyclones and climate change. Wiley Interdiscip Rev Clim Chang. John Wiley & Sons, Inc.; 2016;7: 65–89. doi:10.1002/wcc.371

15. Ivins ER, Dokka RK, Blom RG. Post-glacial sediment load and subsidence in coastal Louisiana. Geophys Res Lett. 2007;34: 1–5. doi:10.1029/2007GL030003

16. NOAA. Sea Level Variations of the United States 1854-2006. Silver Spring, Maryland; 2009.

17. Gesch D, Oimoen M, Greenlee S, Nelson C, Steuck M, Tyler D. The national elevation dataset. Photogramm Eng Remote Sensing. ASPRS AMERICAN SOCIETY FOR PHOTOGRAMMETRY AND; 2002;68: 5–32.

18. NOAA. Explore Coastal Communities, Economy, and Ecosystems The Gulf of Mexico at a Glance: A Second Glance. Washington DC; 2011.

19. USCensus. Coastline Population Trends in the United States: 1960 to 2008. Population Estimates and Projections. [Internet]. 2010. Available: http://www.census.gov/prod/2010pubs/p25-1139.pdf

20. PWC-Economics. World in 2050. The BRICs and beyond: prospects, challenges and opportunities. 2013.

21. NAS. National Academy of Sciences - Water Science and Technology Board and Ocean Studies Board [Internet]. National Academic of Sciences; 2014. Available: http://dels.nas.edu/wstb

22. Aerts JCJH, Botzen WJW, Emanuel K, Lin N, Moel H De. Evaluating Flood Resilience Strategies for Coastal Megacities. Science (80- ). 2014;344: 473–475. doi:10.1126/science.1248222

23. Aerts JCJH, Lin N, Botzen W, Emanuel K, de Moel H. Low-probability flood risk modeling for New York City. Risk Anal. 2013;33: 772–88. doi:10.1111/risa.12008

24. Broekx S, Smets S, Liekens I, Bulckaen D, Nocker L. Designing a long-term flood risk management plan for the Scheldt estuary using a risk-based approach. Nat Hazards. 2010;57: 245–266. doi:10.1007/s11069-010-9610-x

25. Hinkel J, Nicholls RJ, Tol RSJ, Wang ZB, Hamilton JM, Boot G, et al. A global analysis of erosion of sandy beaches and sea-level rise: An application of DIVA. Glob Planet Change. Elsevier B.V.; 2013;111: 150–158. doi:10.1016/j.gloplacha.2013.09.002

26. McFadden L, Nicholls RJ, Vafeidis A, Tol RSJ. A methodology for modeling coastal space for global assessment. 2007; 911–920. doi:10.2112/04-0365.1

27. Cobell Z, Zhao H, Roberts HJ, Clark FR, Zou S. Surge and Wave Modeling for the Louisiana 2012 Coastal Master Plan. J Coast Res. Coastal Education and Research Foundation; 2013; 88–108. doi:10.2112/SI_67_7

28. Fischbach JR, Johnson DR, Ortiz DS, Bryant BG, Hoover M, Ostwald J. Coastal Louisiana Risk Assessment Model. Technical Description and 2012 Coastal Master Plan Analysis Results. 2012.

29. Peyronnin N, Green M, Richards CP, Owens A, Reed D, Chamberlain J, et al. Louisiana ’ s 2012 Coastal Master Plan : Overview of a Science-Based and Publicly Informed Decision-Making Process. J Coast Res. 2013;Sp.Issue: 1–15. doi:10.2112/SI

30. Scawthorn C, Blais N, Seligson H, Tate E, Mifflin E, Thomas W, et al. HAZUS-MH Flood Loss Estimation Methodology. I: Overview and Flood Hazard Characterization. Nat Hazards Rev. 2006;7: 60–71. doi:10.1061/(ASCE)1527-6988(2006)7:2(60)

31. Scawthorn C, Flores P, Blais N, Seligson H, Tate E, Chang S, et al. HAZUS-MH Flood Loss Estimation Methodology. II. Damage and Loss Assessment. Nat Hazards Rev. 2006;7: 72–81. doi:10.1061/(ASCE)1527-6988(2006)7:2(72)

32. Neumann JE, Yohe G, Nicholls RJ, Manion M. Sea-level rise & global climate change: A review of impacts to U.S. coasts. 2000; 1–43.

33. Kirshen P, Merrill S, Slovinsky P, Richardson N. Simplified method for scenario-based risk assessment adaptation planning in the coastal zone. Clim Change. 2012;113: 919–931. doi:10.1007/s10584-011-0379-z

1. <https://github.com/borjagreguero/climada_coastal_hazards_module> [↑](#footnote-ref-2)
