# Supplementary material for: Comparing the cost effectiveness of nature-based and coastal adaptation: A case study from the Gulf Coast of the United States
Source: PLoS One. 2018 Apr 11;13(4):e0192132. doi: 10.1371/journal.pone.0192132 (PMC5894966; doi:10.1371/journal.pone.0192132)
Supplement: S2 File — ‘Permission and sources of photos.doc’. (DOCX) [file pone.0192132.s002.docx]

# Permission and sources of photos.

- Photos in Fig 1: Sources of images: (left and middle) Earth Observatory, National Aeronautics and Space Administration, and (right) National Oceanic and Atmospheric Administration/Department of Commerce, photo credit: Lieut. Commander Mark Moran, NOAA Corps, NMAO/AOC.

Requested permission for NOAA photo:

Saturday, July, 29, to 'photolibrary@noaa.gov'

*I request permission for the open-access journal PLOS ONE to publish* [*https://www.flickr.com/photos/51647007@N08/with/5033310659/*](https://www.flickr.com/photos/51647007@N08/with/5033310659/) *under the Creative Commons Attribution License (CCAL) CC BY 4.0 (*[*http://creativecommons.org/licenses/by/4.0/*](https://protect-us.mimecast.com/s/4QNYBxs008bSn?domain=creativecommons.org)*). Please be aware that this license allows unrestricted use and distribution, even commercially, by third parties. Please reply and provide explicit written permission to publish* [*https://www.flickr.com/photos/51647007@N08/with/5033310659/*](https://www.flickr.com/photos/51647007@N08/with/5033310659/) *under a CC BY license*

Response, July 29:

“*All photos in the NOAA Photo Library are in the public domain with the exception of a very few that are noted in the caption as having some copyright restriction. The NOAA Flickr site is a subset of the NOAA Photo Library. Please feel free to use whatever photo you need for your purposes. If you have questions about images in the future, please provide the image ID which is included in the caption information.*

*Skip Theberge - NOAA Central Library*”

- Photo in Fig 3. Earth Observatory, National Aeronautics and Space Administration.
- Photos in Fig 4.
  - Beach - U.S. Geological Survey, flickr, CC licensed
  - Levee - U.S. Army Corps of Engineers, flickr, CC licensed
  - Floodwall - U.S. Geological Survey, flickr, CC licensed
  - Wetland - U.S. Geological Surve, flickr, CC licensed y
  - Island Barrier - U.S. Geological Survey, Landsat
  - Oyster reefs - U.S. Fish and Wildlife Service, flickr, CC licensed
- Photos in Fig 6
  - Beach - U.S. Geological Survey, flickr, CC licensed
  - Levee - U.S. Army Corps of Engineers, flickr, CC licensed
  - Floodwall - U.S. Geological Survey, flickr, CC licensed
  - Wetland - U.S. Geological Surve, flickr, CC licensed y
  - Island Barrier - U.S. Geological Survey, Landsat
  - Oyster reefs - U.S. Fish and Wildlife Service, flickr, CC licensed
