# Supplementary figures and images for: Comparing the cost effectiveness of nature-based and coastal adaptation: A case study from the Gulf Coast of the United States
Source: PLoS One. 2018 Apr 11;13(4):e0192132. doi: 10.1371/journal.pone.0192132 (PMC5894966; doi:10.1371/journal.pone.0192132)

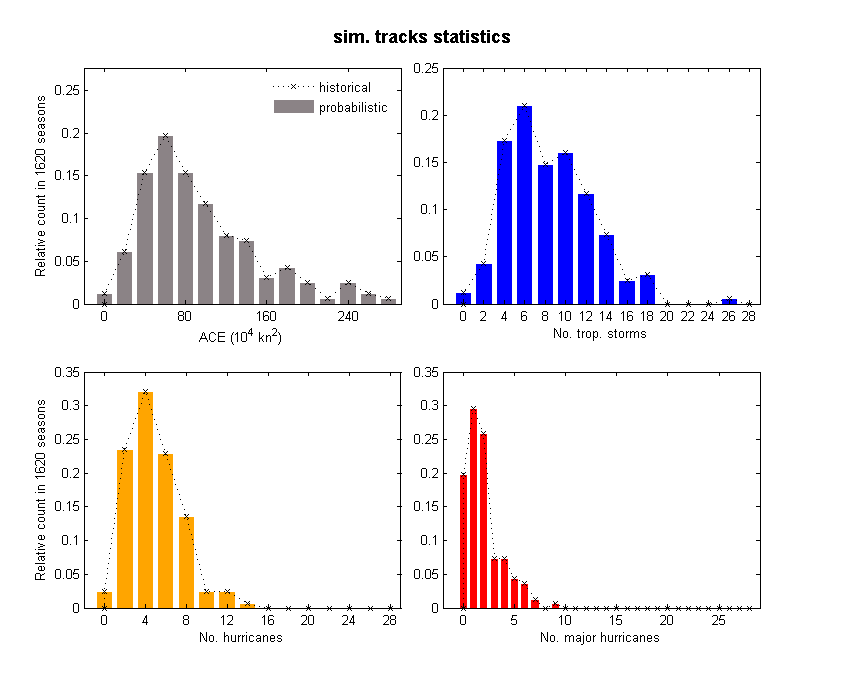

Supplement: S1 Fig — The simulation reproduces the statistical distribution of Accumulated Cyclone Energy (upper left panel), the number of tropical storms (upper right), total number of hurricanes (lower left) and major hurricanes, i.e. category 3 or above (lower right). (PNG) [file pone.0192132.s003.png]

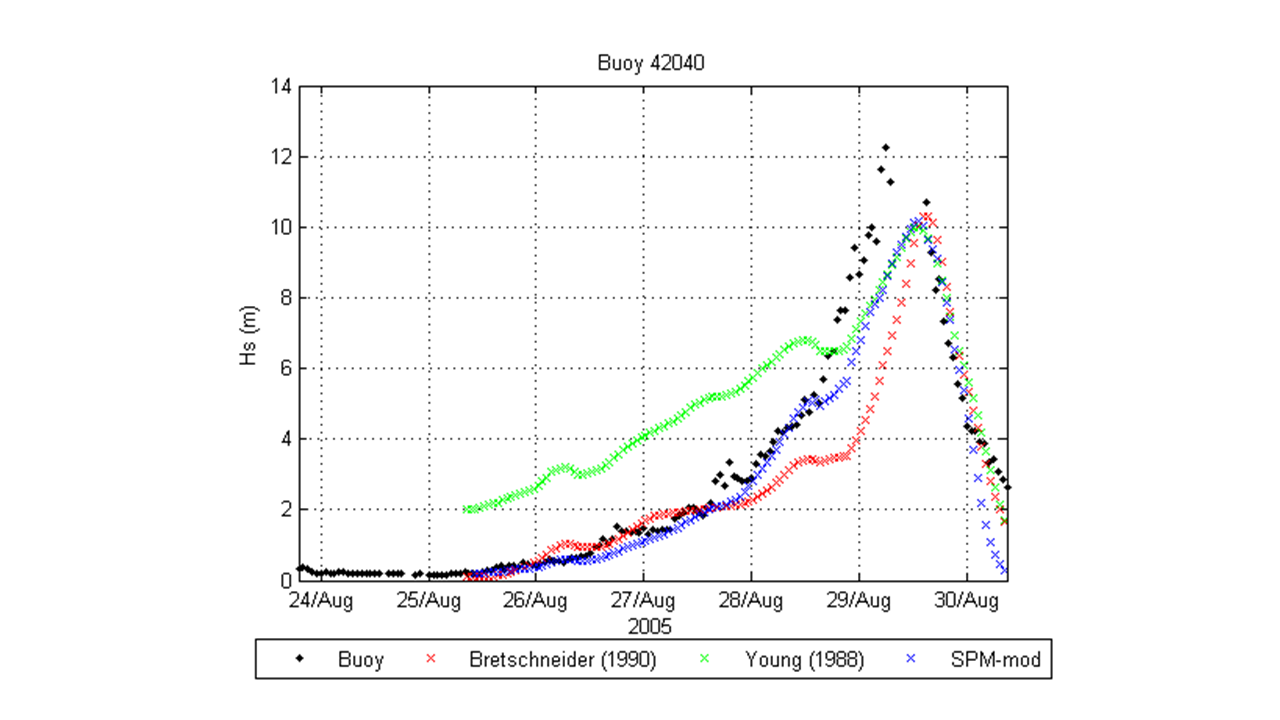

Supplement: S2 Fig — Significant wave height at NOAA buoy 42040 (29.212 North, 88.207 West, 164.6 m deep). (TIF) [file pone.0192132.s004.tif]

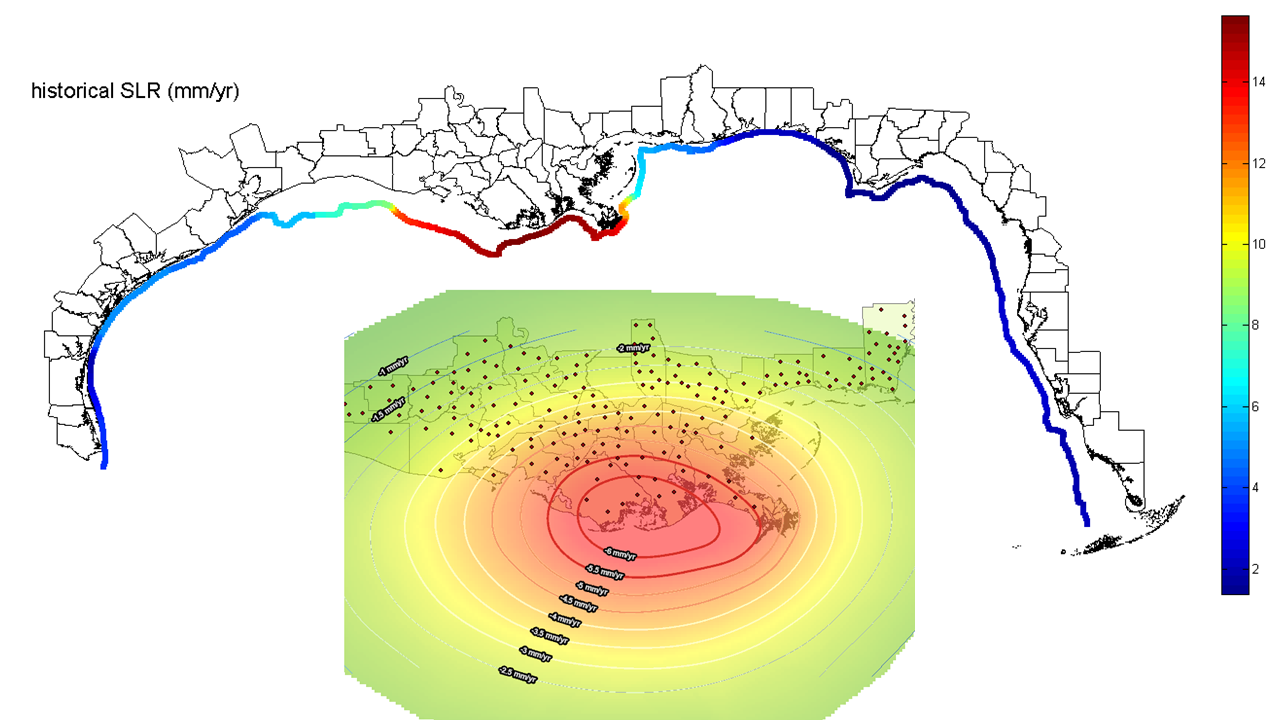

Supplement: S3 Fig — Historical relative Sea Level Rise trends (mm/yr) from NOAA [75] and the subsidence field digitalized from Ivins et al [77]. (TIF) [file pone.0192132.s005.tif]

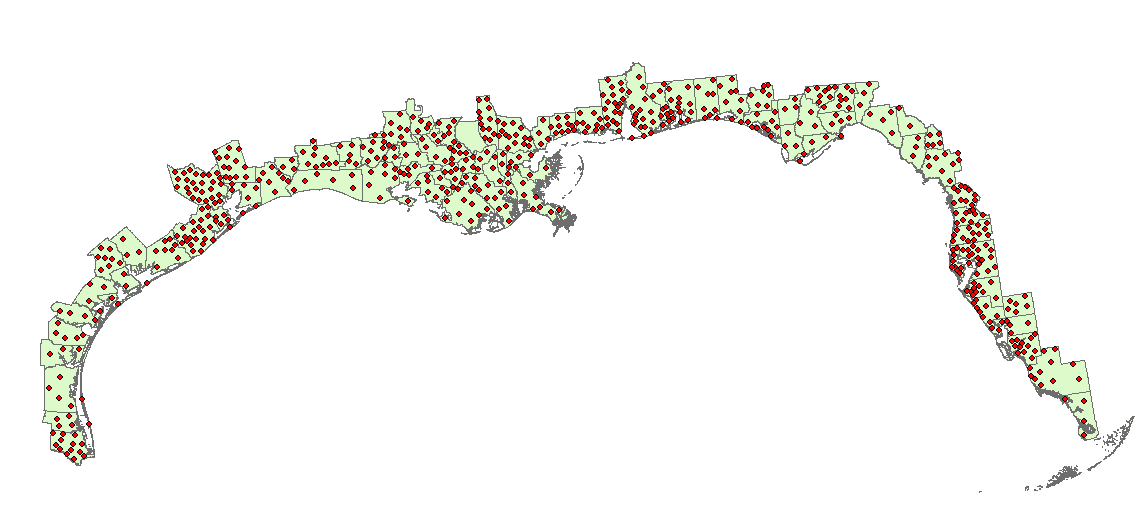

Supplement: S4 Fig — The centroids (red dots) are the basic units where hazards are calculated and then associated to census tracts. (PNG) [file pone.0192132.s006.png]

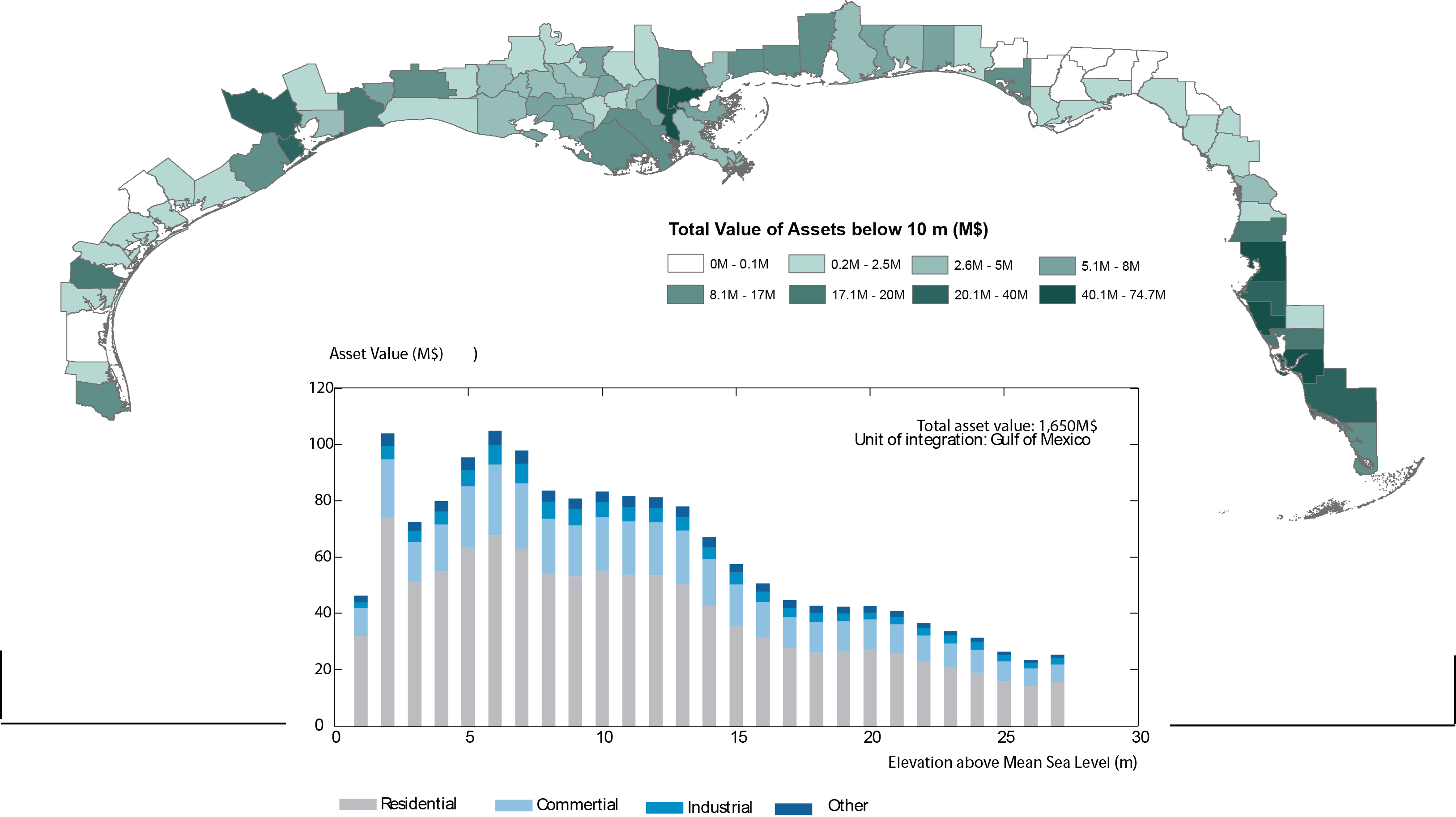

Supplement: S5 Fig — Spatial distribution of value of assets in low-lying zones across the Gulf (below 10 m ground elevation) by county level. The topographic distribution of asset value aggregates for the Gulf is plotted in a subpanel (bar graphic), where the x-axis represents ground height and the y-axis total asset value across the Gulf. (PNG) [file pone.0192132.s007.png]

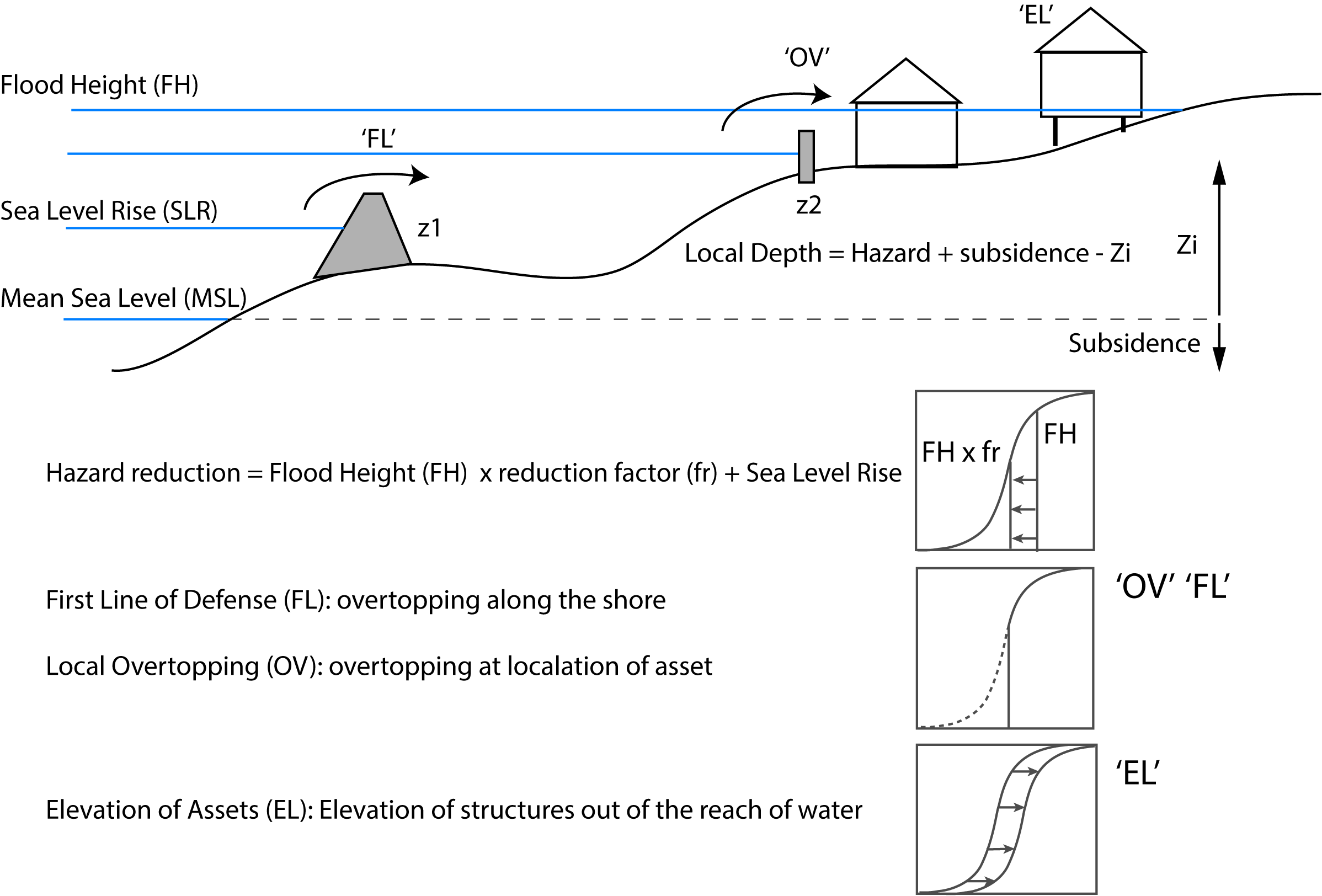

Supplement: S6 Fig — Representation of the model to estimate adaptation for each measure. The S-shape curves represent how adaptation is considered in the damage curves: Hazard Reduction; first line of defense until overtopped (FL); local overtopping (OV); and elevation of structures (EL). MSL: Mean Sea Level, FH: Flooding Height onshore; Zi: topographic elevation at each site (i.e. aggregated at study units). (TIF) [file pone.0192132.s008.tif]

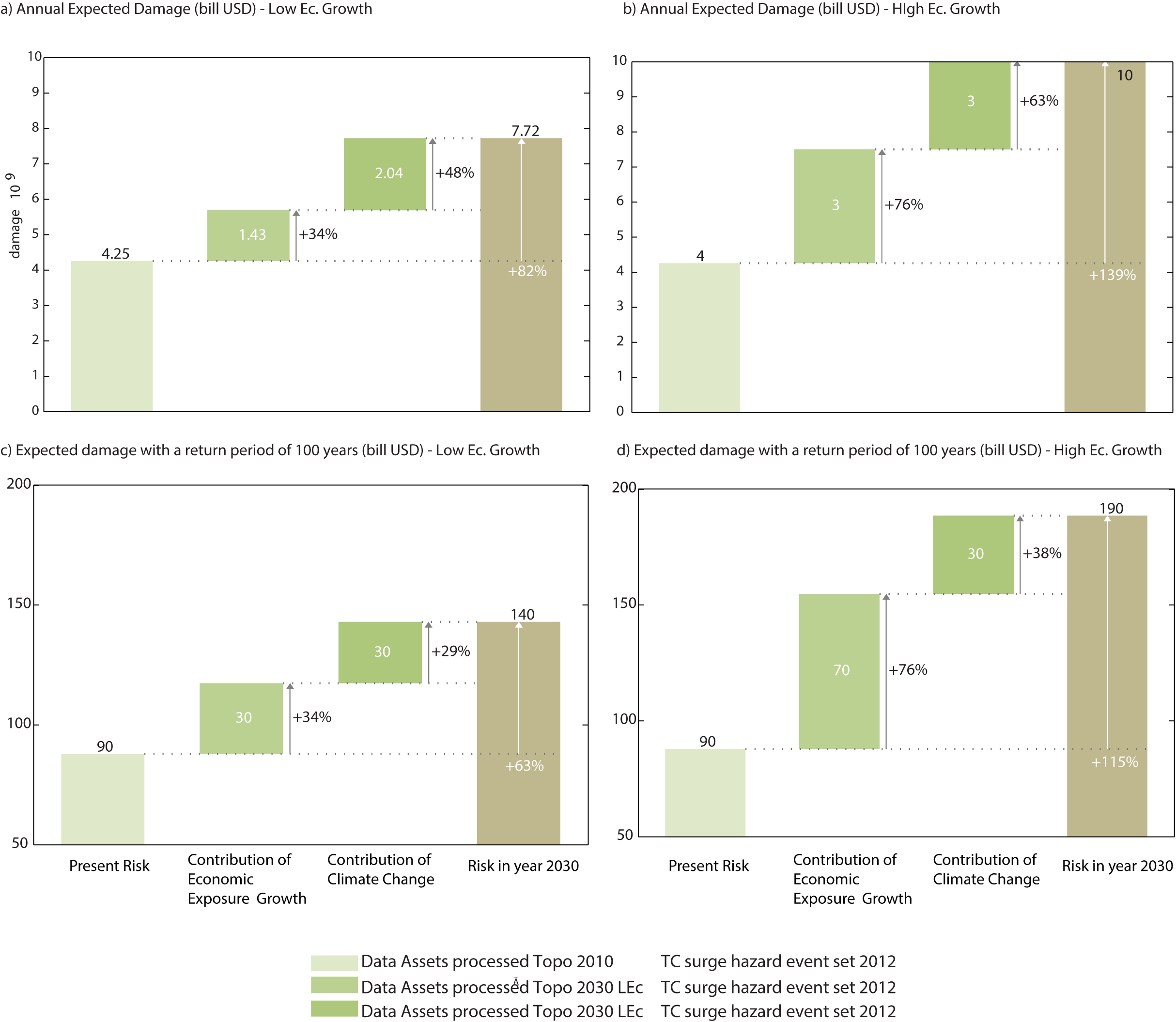

Supplement: S7 Fig — Risk evolution between 2010 and 2030 for the two economic scenarios: Low economic exposure growth (left panels), and high economic exposure Growth (right panels). Bars represent current risk (left) and future risk (right), and separate between the contribution from future economic exposure and climate. Upper panels (a,b) represent the Annual Expected Damage, while the lower panels (c,d) represent the 1-in-100-yr risk. (TIF) [file pone.0192132.s009.tif]

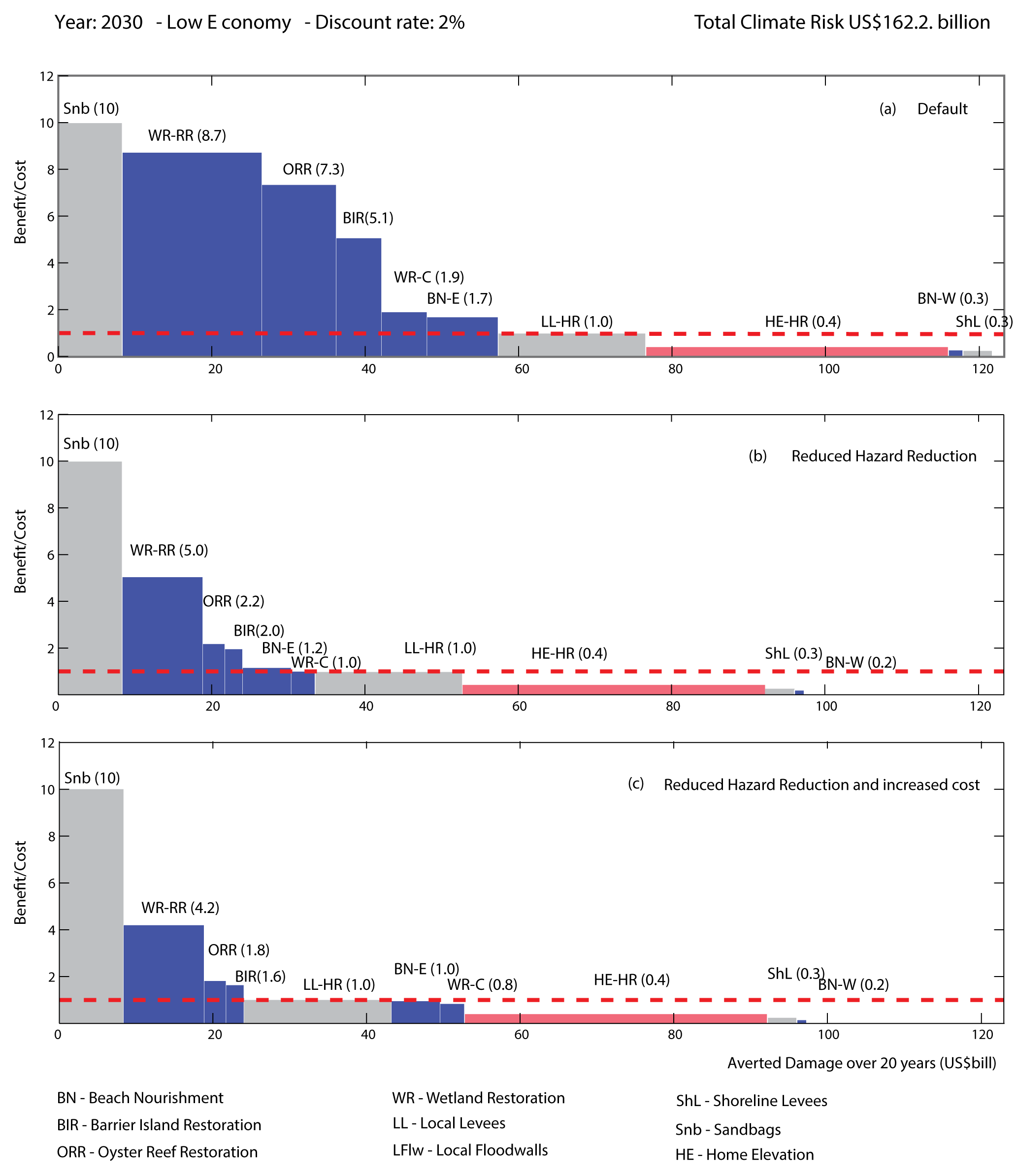

Supplement: S9 Fig — Benefit to cost ratios are represented in the vertical axis (height of the bars), with the horizontal axis noting the aggregated benefit (i.e. total averted damage), and the width of the bars the individual benefit from each measure. Panel a: default estimates of protection and cost, equivalent to Fig 6; Panel b: reduced hazard reduction potential by green options; Panel c: both reduced hazard reduction and increased cost for green options. The scenario corresponds to low discount (2%) and assumes a low economic exposure growth in the next 20 years. (TIF) [file pone.0192132.s011.tif]
