# Supplementary material for: Comparing the cost effectiveness of nature-based and coastal adaptation: A case study from the Gulf Coast of the United States
Source: PLoS One. 2018 Apr 11;13(4):e0192132. doi: 10.1371/journal.pone.0192132 (PMC5894966; doi:10.1371/journal.pone.0192132)
Supplement: S1 Table — Total asset value at reference date (2010) and two future timeframes (2030 and 2050), for the two scenarios of economic exposure growth. (DOCX) [file pone.0192132.s012.docx]

|  | Low Economy (billions USD)  1% compound growth | | High Economy (billions USD)  2% compound growth | |
| --- | --- | --- | --- | --- |
| Total value in 2010 | $ 1,654 | *-* | $ 1,654 | *-* |
| 2030 new value | $ 1,978 | *120%* | $ 2,362 | *142%* |
| 2050 new value | $ 2,462 | *149%* | $ 3,652 | *221%* |

**S1 Table. Present and Future Exposure in the Gulf.** Total asset value at reference date (2010) and two future timeframes (2030 and 2050), for the two scenarios of economic exposure growth.
