# Supplementary material for: Comparing the cost effectiveness of nature-based and coastal adaptation: A case study from the Gulf Coast of the United States
Source: PLoS One. 2018 Apr 11;13(4):e0192132. doi: 10.1371/journal.pone.0192132 (PMC5894966; doi:10.1371/journal.pone.0192132)
Supplement: S2 Table — Range of parameters found in the literature and data sources for attenuation of hazard for different coastal features, along with a brief description of the basic principle for protection. (DOCX) [file pone.0192132.s013.docx]

| TYPE | Principle for Coastal Defense | Hazard reduction (%) | | | | Mechanism of defense used in the protection model | References |
| --- | --- | --- | --- | --- | --- | --- | --- |
|  |  | Short Wave component | | Long Wave component | |  |  |
| Wetland | Reduces storm surge and waves by friction over long distances. Two sizes distinguished. | 60-90 | | 1m / 14.5 km  (1m/ 7 to 20 km)  2 types of wetlands  Size1 – small  Size2 – large | | Hazard reduction depending on size:  Size1-5 km – 0.33-0.5 m  Size2-15 km – 1-2-1.5 m | [1]  [2]  [3] - estimates are in the range of 5 to 40%  *Size 1-5 km – 10%*  *Size 2-15 km – 20%*  [4]  0.1-1 m/km  *Size1–0.5 to 5m*  *Size2 - 1.5 to 15m* |
|  |  |  |  | Size 1  5 - 20 | Size 2  10-30 |  |  |
| Beach and Dunes | Induce breaking of waves and provide a threshold for overtopping | 70 - 90 | |  | | Hazard reduction  Overtopping: low (1m) and high (2m) | [5]  [6] |
| Less resilient beach area (no dunes and narrower) | Urbanized fronts presents less flexibility in reducing wave breaking for high sea levels | 50 | 60 | - | | Hazard reduction | [5]  [6] |
| Island Barrier | Modify the wave propagation pattern and slightly modify the storm surge behind | 50 | 70 | 0 | 10 | Hazard reduction | [7]  [8]  [9] |
| Reefs  (oyster for the Gulf) | Induce breaking of waves. Depend on sea level (i.e. less effective with higher freeboard) | 20^[[1]](#footnote-1)^ | 50 | 0 | 10 | Hazard reduction | [10] |
| Open bay system | Modify the propagation of the Storm Surge inside the bay areas. | 0 | 15 | -10 | 10 | Hazard reduction | Numerical Simulations in bays of the Gulf Coast, USA |
| Coastal Lagoons | Waves do not propagate easily inside. Storm Surge is allowed to penetrate. | 10 | 30 | - | | Hazard reduction | Numerical Simulations in bays of the Gulf Coast, USA |
| Rigid structure | Provides protection based on an overtopping threshold | - | | - | | Overtopping (1m) | Identify in coastal centroids with high concentration of assets  Type 1: 1.5 m (urban hubs)  Type 2: New Orleans Levees (5.3m) |

**S2 Table. Hazard attenuation from different coastal features.** Range of parameters found in the literature and data sources for attenuation of hazard by different coastal features, along with a brief description of the basic principle for protection.

1. Resio DT, Westerink JJ. Modeling the physics of storm surges. Phys Today. 2008;61: 33–38. doi:10.1063/1.2982120

2. Wamsley T V., Cialone M a., Smith JM, Atkinson JH, Rosati JD. The potential of wetlands in reducing storm surge. Ocean Eng. Elsevier; 2010;37: 59–68. doi:10.1016/j.oceaneng.2009.07.018

3. Sheng YP, Lapetina A, Ma G. The reduction of storm surge by vegetation canopies: Three-dimensional simulations. Geophys Res Lett. 2012;39: n/a-n/a. doi:10.1029/2012GL053577

4. Barbier EB, Georgiou IY, Enchelmeyer B, Reed DJ. The Value of Wetlands in Protecting Southeast Louisiana from Hurricane Storm Surges. PLoS One. 2013;8: e58715. doi:10.1371/journal.pone.0058715

5. Stockdon BHF, Doran KJ, Thompson DM, Sopkin KL, Plant NG, Sallenger AH. National Assessment of Hurricane-Induced Coastal Erosion Hazards : Gulf of Mexico. 2012;

6. Stockdon HF, Sallenger Jr. AH, Holman RA, Howd PA. A simple model for the spatially-variable coastal response to hurricanes. Mar Geol. 2007;238: 1–20. doi:http://dx.doi.org/10.1016/j.margeo.2006.11.004

7. Feagin R a., Smith WK, Psuty NP, Young DR, Martínez ML, Carter G a., et al. Barrier Islands: Coupling Anthropogenic Stability with Ecological Sustainability. J Coast Res. 2010;26: 987–992. doi:10.2112/09-1185.1

8. McCall RT, Van Thiel de Vries JSM, Plant NG, Van Dongeren a. R, Roelvink J a., Thompson DM, et al. Two-dimensional time dependent hurricane overwash and erosion modeling at Santa Rosa Island. Coast Eng. Elsevier B.V.; 2010;57: 668–683. doi:10.1016/j.coastaleng.2010.02.006

9. Kindinger JL, Buster N a, Flocks JG, Bernier JC, Kulp M a. Louisiana Barrier Island Comprehensive Monitoring ( BICM ) Program Summary Report : Data and Analyses 2006 through 2010. 2013;

10. Burcharth HF, Hawkins SJ, Zanuttigh B, Lamberti ABT-EDG for LCCS, editors. Summary of the DELOS Project [Internet]. Oxford: Elsevier Science Ltd; 2007. pp. XV–XVIII. doi:10.1016/B978-008044951-7/50020-8

1. variable on sea level [↑](#footnote-ref-1)
