# Supplementary material for: Comparing the cost effectiveness of nature-based and coastal adaptation: A case study from the Gulf Coast of the United States
Source: PLoS One. 2018 Apr 11;13(4):e0192132. doi: 10.1371/journal.pone.0192132 (PMC5894966; doi:10.1371/journal.pone.0192132)
Supplement: S3 Table — Review of cost estimates and sources of information for the definition of the adaptation measures. (DOCX) [file pone.0192132.s014.docx]

| Measure | Unit cost estimate | Supporting References |
| --- | --- | --- |
| Wetland Restoration | Various projects:   - Barataria Bay Waterway Wetland Restoration $2,026,975; 445 acres, $4,560/acre - Sabine Refuge Marsh Creation $5,031,237; 214 acres; $23,510/acre - North Lake Mechant $28,373,125; 902 acres; $31,460/acre - Bayou La Branche Wetland Creation $6,598,109; 203 acres; $32,500/acre - Goose Point/Pointe Platte Marsh Creation $21,049,208; 600 acres; $35,080/acre   Varies between 4,560 to 35,080 $/acre, with an average $12,615,731; 473 acres, $25,422/acre  Louisiana’s Coastal Master Plan – Southwest coast:   - East Rainey marsh creation, 3, 080 acres, 429M$, $139,286/acre - South grand Chenier, 7,330 acres, 708 M$, $96,589/acre - mud lake marsh creation, 3910 acres, 581M$, $148,593/acre - West Rainey, 3,550 acres, 615 M$, $173,239/acre - Southeast Calcasieu Lake, 7,600 acres, 666 M$, $87,632/acre - Cameron Meadows, 3,290 acres, 290 M$, $88,146/acre - East Pecan Island, 7,340 acres, 1180 M$, $160,763/acre - Calcasieu Ship, 2,640 acres, 185 M$, $70,076/acre - East Calcasieu Lake, 14,840 acres, 2,484M$, $167,385/acre - Kelso Bayou, 260 acres, 32M$, $123,077/acre   Average: 125,500$/acre  Louisiana’s Coastal Master Plan – Central coast:   - Terrebonne Bay Rim Marsh Creation Study: 3,370 acres, 91M$, $27,000/acre - Belle Pass Golden Meadow Marsh, 14,420 acres, 732M$, 50,760$/acre - North Terrebonne Bay marsh creation 4,940 acres 155M$, 31,380$/acre - Terrebonne Marsh Creation 1,190 acres 37M$, 31,000$/acre - Belle pass golden meadow (2nd phase), 14,420 acres, 2,927M$, 203,000$/acre   Average: 68,600$/acre (35,000$/acre without Belle Pass)  Louisiana’s Coastal Master Plan – Southeast coast:   - South Lake Lery, 450 acres, 36M$, 80,000$/acre - Hopedale, 550 acres, 147M$. 267,000$/acre - New Orelans East Landbridge Restoration, 8,510 acres, 473M$, 55,580$/acre - Lake Borgne, 2,230 acres, 620M$, 278,000$/acre - Central wetland, 2,010 acres, 234M$, 116,400$/acre - Golden Triangle, 2,440, 392M$, 160,000$/acre - Large scale Barataria, 8,070 acres, 293M$, 36,000$/acre - Grand Liard, 560 acres, 34M$, 6,000$/acre - New Orleans Eastl Landbridge (2nd phase), 8,510 acres, 1,890M$, 222,000$/acre - Biloxi, 33,280 acres, 3,046M$, 91,000$/acre - Large scale Barataria (2nd phase), 8070 acres, 1,980M$, 245,000$/acre - Barataria Bay Rim, 2,010 acres, 216M$, 107,460$/acre   Average: 138,700$/acre | [1]  [2]  [3] and references therein |
| Local Levees | Levees:  2 feet above ground level – $39 per linear foot  4 feet above ground level – $73 per linear foot  6 feet above ground level – $122 per linear foot | [4] |
| Sandbags | Retail Cost Pre-Filled Sandbags:  Average cost of a pre-filled 50 lbs sandbag = $2.25 (shipping/delivery and placement around structure not included)  Polypropylene Olive Drab Sandbag retails for $3 to $6  3$ in Amazon  Example: boxbarrier:  134.6250 $/m (0.5 m high)  60.7500 for sandbags (30 sandbags/m) | [4]  <http://barriersystemsllc.com/>  <http://www.howmuchisit.org/how-much-do-sandbags-cost/>  Boxbarrier: <http://www.boxbarrier.com/our-product/costs/item27> |
| Local Floodwalls | A concrete block wall costs $6.10 to $7.60 per linear foot.  Floodwalls, 2 feet above ground level - $ 77 per linear foot  Floodwalls, 4 feet above ground level -$113 per linear foot  Floodwalls, 6 feet above ground level - $160 per linear foot  + 10% benefit contractor + update prices from 1993 | [4]  [www.homeadvisor.com](http://www.homeadvisor.com) |
| Levees | - 10,000$/ft (24 feet high) - Morganza: 98-mile-long, $10.3 billion - 24 feet high – with hurricane Katrina (2007) standards and a 20% reduction in cost from economy of scale - $4,000 to $8,000 per linear foot   Louisiana’s coastal master plan:   - Morganza to the Gulf, levee to an elevation of 19.6-36.5 ft, 60.4 miles, 3,964M$, 65.6M$/ml, 11,720$/linear foot - Morgan city back levee, 13.5 ft high, 8 miles, 257M$, 32M$/mile, 6,400$/linear foot - Abbeville and Vicinity, 17-20 ft high, 39.4 miles, 958M$, 24M$/ml, 4,600$/linear foot - Berwick to Wax Lake, 18ft high, 72,000 feet, 253M$, 3,500$/linear foot - Franklin and vicinity, 16.5 ft high, 285,000 feet, with pumps, 975M$, 3,420$/linear foot - Greater New Orleans LaPlace Extension, 13.5 ft high, 140,000 ft, 457M$, 3,400$/ linear foot - Slidell Rink Levee, 16 ft high, 36,000 ft, 81M$, 2,250$/ linear foot - Lafitte Ring Levee, 16 ft high, 156,000 ft and flood gates and pumps, 870M$, 5600$/ linear foot - Maintain West Bank levees, 145,000 ft, 193M$, 1330 $/ linear foot | [4]  Review of USACE – projects  [5]  <http://www.stcplanning.org/>  <http://www.stronglevees.com/cost/>  [2]  [3] and references therein |
| Barrier Island Restoration | - Shell Island project: 36.6mill$/ml - Pelican Island project: 64,200$/acre - Plaquemines Parish: 14.6mill$/ml - Scofield project: 72,630$/acre   $17,000 to $102,000/acre. Average: $51,300/acre  Louisiana’s coastal master plan:   - Barataria Pass to Sandy Point, storm surge reduction and wave attenuation, 535M$, unknown surface - Belle Pass to Caminada Pass, storm surge reduction and wave attenuation, 281M$, unknown surface | [6]  [1]  [2]  [3] and references therein |
| Oyster Reef Restoration | - $1.5 million / mile   Louisiana’s Coastal Master Plan   - West Cote Blanche bay oyster barrier reef restoration, 28,000 ft, 20M$, 3.7M$/ml - East Cote Balnche bay oyster barrier reef restoration, 30,000 ft, 22M$, 3.8M$/ml | [7]  [8,9]  [2]  [3] and references therein |
| Beach Nourishment and restoration | - $4.5 million per mile nourished - $6.5 cubic yard after revising USACE (2003), assuming and additional cost of 10% for operations and another 10% for maintenance.   Prices are updated to 2010 values using the Civil Works Construction Cost Index System (CWCCIS) guidance [10] | [11]  [12]  [11]  <http://beachnourishment.wcu.edu/about.php>  Florida Beach Nourishment Projects Monitoring Database: <http://beach15.beaches.fsu.edu/> |
| Home Elevation of Existing Homes in areas of High Priority for reducing risk | Elevation:   - 2-foot raise: Wood frame building with basement or crawlspace – $18 per square foot   Other features:   - Wood frame building with slab-on-grade foundation – $50 per square foot - Masonry building with basement or crawlspace – $37 per square foot - Masonry building with slab-on-grade foundation – $50 per square foot - 3 to 8 foot raise – add $0.80 per square foot for each additional foot of elevation - Above 8 feet – add $1.05 per square foot - Wood frame with brick veneer on walls – add 10% | Gulf projects and personal communication with contractors |

**S3 Table. Cost estimates and sources of information.** Review of cost estimates and sources of information for the definition of the adaptation measures.

**References**

1. Thomson G, Ce D, Brodnax C. Barrier Island Restoration in the Gulf Coast - Is it Worth it ? 2010; Available: https://www.estuaries.org/pdf/2010conference/monday15/yacht/session3/thomson-brodnax.pdf

2. Louisiana CP and RA of L. Louisiana’s Comprehensive Master Plan for a Sustainable Coast [Internet]. Baton Rouge; 2012. Available: https://issuu.com/coastalmasterplan/docs/coastal_master_plan-v2

3. CPRA. Louisiana’s Comprehensive Master Plan for a Sustainable Coast [Internet]. State of Louisiana.; 2012. Available: https://biotech.law.lsu.edu/la/coast/2012-Coastal-Master-Plan.pdf

4. FEMA. Homeowner’s Guide to Retrofitting [Internet]. 2014. Available: http://www.fema.gov/floodproofing

5. USACE. North Atlantic Coast Comprehensive Study: Resilient Adaptation to Increasing Risk [Internet]. 2015. Available: http://www.nad.usace.army.mil/Portals/40/docs/NACCS/NACCS_main_report.pdf

6. Kindinger JL, Buster N a, Flocks JG, Bernier JC, Kulp M a. Louisiana Barrier Island Comprehensive Monitoring ( BICM ) Program Summary Report : Data and Analyses 2006 through 2010. 2013;

7. Kroeger T. Dollars and Sense : Economic Benefits and Impacts from two Oyster Reef Restoration Projects in the Northern Gulf of Mexico The Nature Conservancy. The Nature Conservancy. 2012.

8. DePiper G, Lipton D. Payment for Ecosystem Services From Oyster Reef Restoration : Possibilities for Chesapeake Bay. 2011;

9. Kroeger T. Oyster reef restoration in the northern Gulf of Mexico: Assessing economic rationales for large-scale restoration efforts. TEEB 2012 Conf March 2012, Leipzig, Ger. 2012; 1–47.

10. USACE. Civil Works Construction Cost Index System (CWCCIS) - EM 1110-2-1304. 2012.

11. Trembanis AC, Pilkey OH. Comparison of Beach Nourishment along the U.S. Atlantic, Great Lakes, Gulf of Mexico, and New England Shorelines. Coast Manag. 1999;27: 329–340. doi:10.1080/089207599263730

12. USACE. THE CORPS OF ENGINEERS AND SHORE PROTECTION • History • Projects • Costs i. 2003;
