# Supplementary material for: Comparing the cost effectiveness of nature-based and coastal adaptation: A case study from the Gulf Coast of the United States
Source: PLoS One. 2018 Apr 11;13(4):e0192132. doi: 10.1371/journal.pone.0192132 (PMC5894966; doi:10.1371/journal.pone.0192132)
Supplement: S4 Table — Benefit to cost ratios and total benefit for the list of adaptation measures, across scenarios of costs and effectiveness. The total climate risk for the scenario is 176.6 US$ billion (calculated from Annual Expected Damages over a 20-year period). Total cost-effective adaptation is the aggregated value of the total benefits (TB) for all measures with benefit to coast ratio (B/C) above 1. The nature-based cost-effective adaptation is the aggregated value of the total benefits only for the nature-based measures (wetland restoration, beach nourishment, barrier island restoration, and oyster reef restoration) with a benefit to cost ratio above 1. Values correspond to the high economic exposure growth for the year 2030. The discount rate of benefits and costs is 2%. In columns, the ‘more conservative’ and ‘most conservative’ cost-benefit scenarios correspond to (see Methods): the sensitivity analysis where the effectiveness of green measures are reduced and the costs increased by 20% (costs are only modified in the most conservative scenario). (DOCX) [file pone.0192132.s015.docx]

| Scenario: Year 2030- High Economy, Discounting = 2% | | DEFAULT | | SENSITIVITY of effectiveness | | CONSERVATIVE in effectiveness and cost | |
| --- | --- | --- | --- | --- | --- | --- | --- |
| MEASURE NAME | | B/C | TB | B/C | TB | B/C | TB |
| Local Levees | | 1.31 | 25.3 | 1.31 | 25.3 | 1.31 | 25.3 |
| Sandbags | | 13.18 | 11.1 | 13.18 | 11.1 | 13.18 | 11.1 |
| Dykes & Levees | | 0.34 | 5.1 | 0.34 | 5.1 | 0.34 | 5.1 |
| Home Elevation | | 0.53 | 52.0 | 0.53 | 52.0 | 0.53 | 52.0 |
| Wetland Restoration | Risk Reduction Priority | 11.49 | 24.0 | 6.64 | 13.8 | 5.53 | 13.8 |
|  | Conservation Priority | 2.51 | 7.8 | 1.32 | 4.1 | 1.10 | 4.1 |
| Barrier Island Restoration | | 6.68 | 7.8 | 2.58 | 3.0 | 2.15 | 3.0 |
| Oyster Reef Restoration | | 9.68 | 12.7 | 2.86 | 3.8 | 2.39 | 3.8 |
| Beach nourishment | western Gulf (TX) | 0.36 | 2.5 | 0.25 | 1.7 | 0.21 | 1.7 |
|  | eastern Gulf (FL) | 2.21 | 12.2 | 1.52 | 8.4 | 1.27 | 8.4 |
| *Total cost-effective adaptation* | |  | *101.0* |  | *69.5* |  | *69.5* |
| *Total Nature-based cost-effective adaptation* | |  | *64.6* |  | *33.1* |  | *33.1* |

**S4 Table. Benefit to cost ratios for year 2030 under a high economic exposure growth.** Benefit to cost ratios and total benefit for the list of adaptation measures, across scenarios of costs and effectiveness. The total climate risk for the scenario is 176.6 US$ billion (calculated from Annual Expected Damages over a 20-year period). Total cost-effective adaptation is the aggregated value of the total benefits (TB) for all measures with benefit to coast ratio (B/C) above 1. The nature-based cost-effective adaptation is the aggregated value of the total benefits only for the nature-based measures (wetland restoration, beach nourishment, barrier island restoration, and oyster reef restoration) with a benefit to cost ratio above 1. Values correspond to the high economic exposure growth for the year 2030. The discount rate of benefits and costs is 2%. In columns, the ‘more conservative’ and ‘most conservative’ cost-benefit scenarios correspond to (see Methods): the sensitivity analysis where the effectiveness of green measures are reduced and the costs increased by 20% (costs are only modified in the most conservative scenario).
