# Supplementary material for: Comparing the cost effectiveness of nature-based and coastal adaptation: A case study from the Gulf Coast of the United States
Source: PLoS One. 2018 Apr 11;13(4):e0192132. doi: 10.1371/journal.pone.0192132 (PMC5894966; doi:10.1371/journal.pone.0192132)
Supplement: S5 Table — Benefit to cost ratios and total benefit for the list of adaptation measures, across scenarios of costs and effectiveness. The total climate risk for the scenario is 398.2 US$ billion (calculated from Annual Expected Damages over a 40-year period). Total cost-effective adaptation is the aggregated value of the total benefits (TB) for all measures with benefit to coast ratio (B/C) above 1. The nature-based cost-effective adaptation is the aggregated value of the total benefits only for the nature-based measures (wetland restoration, beach nourishment, barrier island restoration, and oyster reef restoration) with a benefit to cost ratio above 1. Values correspond to the low economic growth for the year 2050. The discount rate of benefits and costs is 2%. In columns, the ‘more conservative’ and ‘most conservative’ cost-benefit scenarios correspond to (see Methods): the sensitivity analysis where the effectiveness of green measures are reduced and the costs increased by 20% (costs are only modified in the most conservative scenario). (DOCX) [file pone.0192132.s016.docx]

| Scenario: Year 2050 – Low Economy. – Intermediate (Low) defense – Discounting = 2% | | DEFAULT | | SENSITIVITY of effectiveness | | CONSERVATIVE in effectiveness and cost | |
| --- | --- | --- | --- | --- | --- | --- | --- |
| MEASURE NAME | | B/C | TB | B/C | TB | B/C | TB |
| Local Levees | | 3.01 | 58.4 | 3.01 | 58.4 | 3.01 | 58.4 |
| Sandbags | | 28.72 | 24.1 | 28.72 | 24.1 | 28.72 | 24.1 |
| Dykes & Levees | | 0.95 | 14.2 | 0.95 | 14.2 | 0.95 | 14.2 |
| Home Elevation | | 1.14 | 110.9 | 1.14 | 110.9 | 1.14 | 110.9 |
| Wetland Restoration | Risk Reduction Priority | 28.52 | 59.5 | 16.77 | 35.0 | 13.97 | 35.0 |
|  | Conservation Priority | 6.28 | 19.6 | 3.35 | 10.5 | 2.79 | 10.5 |
| Barrier Island Restoration | | 18.94 | 22.1 | 8.59 | 10.0 | 7.16 | 10.0 |
| Oyster Reef Restoration | | 29.07 | 38.2 | 12.28 | 16.1 | 10.23 | 16.1 |
| Beach nourishment | western Gulf (TX) | 1.46 | 9.9 | 1.05 | 7.2 | 0.88 | 7.2 |
|  | eastern Gulf (FL) | 8.45 | 46.7 | 5.97 | 33.0 | 4.97 | 33.0 |
| *Total Cost Effective* | |  | *389.4* |  | *305.1* |  | *297.9* |
| *Total Nature-based cost effective measures* | |  | *186.1* |  | *111.7* |  | *104.6* |

**S5 Table. Benefit to cost ratios for year 2050 under a low economic exposure growth**. Benefit to cost ratios and total benefit for the list of adaptation measures, across scenarios of costs and effectiveness. The total climate risk for the scenario is 398.2 US$ billion (calculated from Annual Expected Damages over a 40-year period). Total cost-effective adaptation is the aggregated value of the total benefits (TB) for all measures with benefit to coast ratio (B/C) above 1. The nature-based cost-effective adaptation is the aggregated value of the total benefits only for the nature-based measures (wetland restoration, beach nourishment, barrier island restoration, and oyster reef restoration) with a benefit to cost ratio above 1. Values correspond to the low economic growth for the year 2050. The discount rate of benefits and costs is 2%. In columns, the ‘more conservative’ and ‘most conservative’ cost-benefit scenarios correspond to (see Methods): the sensitivity analysis where the effectiveness of green measures are reduced and the costs increased by 20% (costs are only modified in the most conservative scenario).
