# Supplementary material for: Comparing the cost effectiveness of nature-based and coastal adaptation: A case study from the Gulf Coast of the United States
Source: PLoS One. 2018 Apr 11;13(4):e0192132. doi: 10.1371/journal.pone.0192132 (PMC5894966; doi:10.1371/journal.pone.0192132)
Supplement: S6 Table — Benefit to cost ratios and total benefit for the list of adaptation measures, across scenarios of costs and effectiveness. Total cost-effective adaptation is the aggregated value of the total benefits (TB) for all measures with benefit to coast ratio (B/C) above 1. The nature-based cost-effective adaptation is the aggregated value of the total benefits only for the nature-based measures (wetland restoration, beach nourishment, barrier island restoration, and oyster reef restoration) with a benefit to cost ratio above 1. Values correspond to the low economic growth for the year 2030. The cost-benefit scenario corresponds to the default setting. (DOCX) [file pone.0192132.s017.docx]

| Year 2030 – Low Economy – Intermediate (low) defense – Default parameters of performance | | Discount rate = 2% | | Discount rate = 10% | |
| --- | --- | --- | --- | --- | --- |
| MEASURE NAME | | B/C | TB | B/C | TB |
| Local Levees | | 0.99 | 19.2 | 0.54 | 10.5 |
| Sandbags | | 10.00 | 8.4 | 5.48 | 4.6 |
| Dykes & Levees | | 0.26 | 3.9 | 0.14 | 2.1 |
| Home Elevation | | 0.41 | 39.4 | 0.22 | 21.6 |
| Wetland Restoration | Risk Reduction Priority | 8.72 | 18.2 | 4.78 | 10.0 |
|  | Conservation Priority | 1.90 | 5.9 | 1.04 | 3.3 |
| Barrier Island Restoration | | 5.07 | 5.9 | 2.78 | 3.2 |
| Oyster Reef Restoration | | 7.34 | 9.7 | 4.03 | 5.3 |
| Beach nourishment | Western Gulf (TX) | 0.28 | 1.9 | 0.15 | 1.0 |
|  | Eastern Gulf (FL) | 1.68 | 9.3 | 0.92 | 5.1 |
| *Total Cost Effective* | |  | *57.3* |  | *26.4* |
| *Total NbD cost effective* | |  | *49.0* |  | *21.8* |

**S6 Table. Effect of discounting rates.** Benefit to cost ratios and total benefit for the list of adaptation measures, across scenarios of costs and effectiveness. Total cost-effective adaptation is the aggregated value of the total benefits (TB) for all measures with benefit to coast ratio (B/C) above 1. The nature-based cost-effective adaptation is the aggregated value of the total benefits only for the nature-based measures (wetland restoration, beach nourishment, barrier island restoration, and oyster reef restoration) with a benefit to cost ratio above 1. Values correspond to the low economic growth for the year 2030. The cost-benefit scenario corresponds to the default setting.
